# Profiling hippocampal neuronal populations reveals unique gene expression mosaics reflective of connectivity-based degeneration in the Ts65Dn mouse model of Down syndrome and Alzheimer’s disease

**DOI:** 10.3389/fnmol.2025.1546375

**Published:** 2025-02-26

**Authors:** Melissa J. Alldred, Kyrillos W. Ibrahim, Harshitha Pidikiti, Sang Han Lee, Adriana Heguy, Gabriela Chiosis, Elliott J. Mufson, Grace E. Stutzmann, Stephen D. Ginsberg

**Affiliations:** ^1^Center for Dementia Research, Nathan Kline Institute, Orangeburg, NY, United States; ^2^Department of Psychiatry, New York University Grossman School of Medicine, New York, NY, United States; ^3^Genome Technology Center, New York University Grossman School of Medicine, New York, NY, United States; ^4^Program in Chemical Biology, Sloan Kettering Institute, New York, NY, United States; ^5^Breast Cancer Medicine Service, Memorial Sloan Kettering Cancer Center, New York, NY, United States; ^6^Department of Translational Neuroscience and Neurology, Barrow Neurological Institute, Phoenix, AZ, United States; ^7^Center for Neurodegenerative Disease and Therapeutics, Rosalind Franklin University, The Chicago Medical School, North Chicago, IL, United States; ^8^Department of Neuroscience and Physiology, New York University Grossman School of Medicine, New York, NY, United States; ^9^NYU Neuroscience Institute, New York University Grossman School of Medicine, New York, NY, United States

**Keywords:** Alzheimer’s disease, bioinformatics, Down syndrome, hippocampus, laser capture microdissection, RNA sequencing, selective vulnerability, trisomy

## Abstract

**Introduction:**

Individuals with Down syndrome (DS) exhibit neurological deficits throughout life including the development of in Alzheimer’s disease (AD) pathology and cognitive impairment. At the cellular level, dysregulation in neuronal gene expression is observed in postmortem human brain and mouse models of DS/AD. To date, RNA-sequencing (RNA-seq) analysis of hippocampal neuronal gene expression including the characterization of discrete circuit-based connectivity in DS remains a major knowledge gap. We postulate that spatially characterized hippocampal neurons display unique gene expression patterns due, in part, to dysfunction of the integrity of intrinsic circuitry.

**Methods:**

We combined laser capture microdissection to microisolate individual neuron populations with single population RNA-seq analysis to determine gene expression analysis of CA1 and CA3 pyramidal neurons and dentate gyrus granule cells located in the hippocampus, a region critical for learning, memory, and synaptic activity.

**Results:**

The hippocampus exhibits age-dependent neurodegeneration beginning at ~6 months of age in the Ts65Dn mouse model of DS/AD. Each population of excitatory hippocampal neurons exhibited unique gene expression alterations in Ts65Dn mice. Bioinformatic inquiry revealed unique vulnerabilities and differences with mechanistic implications coinciding with onset of degeneration in this model of DS/AD.

**Conclusions:**

These cell-type specific vulnerabilities may underlie degenerative endophenotypes suggesting precision medicine targeting of individual populations of neurons for rational therapeutic development.

## Introduction

Down syndrome (DS), caused by the triplication of human chromosome 21 (HSA21), represents the most common genomic cause of intellectual disability with prevalence estimates of 1 in 700 live births in the United States (Mai et al., 2019; Parker et al., 2010). Individuals with DS have multiple neurodevelopmental phenotypes, mild to moderate intellectual impairment, neurological deficits, including reduced numbers and size of neurons in the hippocampus, along with systemic peripheral deficits (Rachidi and Lopes, 2008; Chapman and Hesketh, 2000; Lott, 2012; Wisniewski, 1990; Freeburn and Munn, 2021; Emili et al., 2024). DS results in memory deficits associated with impaired hippocampal function, including impairment in episodic and spatial memory (Freeburn and Munn, 2021; Chapman and Hesketh, 2000; Contestabile et al., 2017; Das et al., 2014). In addition, individuals with DS develop amyloid-beta peptide (Aβ) senile plaques, tau-containing neurofibrillary tangles, cortical thinning, and overt brain atrophy over their lifespan (Chapman and Hesketh, 2000; Mann et al., 1984; Lott and Head, 2019; Wisniewski et al., 1985). Most individuals with DS have documented progressive cognitive impairment, with dementia now described as the primary cause of death in adults with DS (Lott and Head, 2019; Landes et al., 2020).

Murine trisomic models of DS allow researchers to explore neuronal degenerative phenotypes at specific aging timepoints, linking them to the human condition. The Ts65Dn model is one of the oldest and most popular models of DS and AD (DS/AD) (Reeves et al., 1995; Davisson et al., 1993). Ts65Dn mice have a freely segregating mini-chromosome which encompasses a partial triplication of HSA21 orthologs on mouse chromosome 16 (Mmu16; ~90 protein-coding genes), along with a centromeric segment of non-orthologous mouse chromosome 17 (Mmu17; segment 17q1a) (Duchon et al., 2011; Sturgeon and Gardiner, 2011; Akeson et al., 2001). Ts65Dn mice recapitulate many of the endophenotypes associated with human DS, including early endosomal abnormalities (Cataldo et al., 2003; Cataldo et al., 2000), age-associated behavioral deficits in multiple domains of cognition (Powers et al., 2016; Hunter et al., 2003a; Hyde and Crnic, 2001; Fernandez and Garner, 2008), and frank neuronal loss (Gautier et al., 2023; Ash et al., 2014; Kelley et al., 2014b; Velazquez et al., 2013). Cognitive decline in DS has been associated with degeneration of the cholinergic septohippocampal pathway, arising from neuronal loss in the basal forebrain and loss of cholinergic fiber projection to the hippocampus and neocortex, which occurs in the Ts65Dn model of DS/AD (Perez et al., 2019; Velazquez et al., 2013; Powers et al., 2016; Peng et al., 2009; Kelley et al., 2014b; Hamlett et al., 2016; Gautier et al., 2023; Strupp et al., 2016). Moreover, Ts65Dn mice display reduced hippocampal neurogenesis (Bianchi et al., 2010; Velazquez et al., 2013; Insausti et al., 1998), hippocampal synapse loss (Kurt et al., 2004; Popov et al., 2011), and synaptic structural abnormalities (Belichenko et al., 2007; Belichenko et al., 2004; Kleschevnikov et al., 2012a). Degeneration of the cholinergic septohippocampal circuit, a cardinal feature of DS and AD (Granholm et al., 2000; Whitehouse et al., 1982; Yates et al., 1980), starts at approximately 6 months of age (MO) in the Ts65Dn mouse model (Rueda et al., 2012; Gotti et al., 2011; Holtzman et al., 1996). However, the underlying cellular mechanisms driving degeneration of this circuit are understudied.

Previous hippocampal analysis of synaptic structural and functional alterations in DS have linked impaired plasticity as a causal agent of cognitive decline (Kleschevnikov et al., 2004; Costa and Grybko, 2005; Kurt et al., 2004). Ts65Dn mice display decreased excitatory neurons and synaptic density (Kurt et al., 2004), and increased inhibitory neurons and synapses in the hippocampus (Chakrabarti et al., 2010; Belichenko et al., 2004). In the CA1 sector of the hippocampus, decreased long-term potentiation (LTP) occurs at 2 MO and 9 MO in Ts65Dn mice (Siarey et al., 1997), while field potential recordings centered in the CA1 region showed increased long-term depression (LTD) in 2MO Ts65Dn mice (Siarey et al., 1999). Increased spontaneous inhibitory postsynaptic current frequency was observed independently in the Ts65Dn CA1 sector, but no difference was observed in miniature IPSCs (mIPSCs) (Chakrabarti et al., 2010). In contrast, neurons in the Ts65Dn CA3 sector of the hippocampus have decreased mIPSCs and miniature excitatory postsynaptic currents (Hanson et al., 2007; Stagni et al., 2013; Contestabile et al., 2017). Electrophysiological analysis of dentate gyrus granule cells (DGCs) revealed increased inhibitory currents and synaptic density along with decreased LTP (Contestabile et al., 2017; Kleschevnikov et al., 2012b; Kleschevnikov et al., 2004; Contestabile et al., 2013). Taken together, these electrophysiological studies confirm an imbalance of excitation and inhibition in the hippocampus, postulated to be causal to the spatial and episodic memory impairments in DS mouse models, substantiated by GABA and NMDA receptor pharmacotherapies (Fernandez et al., 2007; Costa, 2011; Costa et al., 2008). However, most of these studies were performed in juvenile pups, prior to onset of septohippocampal degeneration, with virtually no assessment of the effects of aging on this learning and memory circuit.

Recent RNA sequencing (RNA-seq) analysis of neurons within medial septal nucleus (MSN) by our laboratory revealed transcriptomic alterations in Ts65Dn mice at the start (~6 MO) of basal forebrain cholinergic neuron (BFCN) degeneration (Alldred et al., 2021c; Alldred et al., 2023). However, hippocampal gene expression studies in postmortem human DS and DS/AD mouse models have mostly been limited to microarray and RT-qPCR based analyses (Alldred et al., 2018; Alldred et al., 2015a; Alldred et al., 2015b; Ahmed et al., 2012; Bofill-De Ros et al., 2015; Granholm et al., 2003; Hunter et al., 2003b; Pollonini et al., 2008). Although a few RNA-seq studies have been performed, most examined the entire hippocampus (Zhou et al., 2023; Granno et al., 2019; Hu et al., 2022), with limited assessment of individual hippocampal subregions in DS/AD models (Alldred et al., 2024a; Sierra et al., 2024). Both our study of laser capture microdissection (LCM) microisolated CA1 pyramidal neurons in ~11 MO female DS mice using the Ts2 derivative of the Ts65Dn model and the single nucleus RNA-seq performed on multiple hippocampal neuronal subtypes exhibited fewer dysregulated genes and pathways compared to the 6 MO MSN BFCNs (Alldred et al., 2024a; Sierra et al., 2024), leading to the hypothesis that (*i*) hippocampal neuronal degeneration lags behind that seen in basal forebrain and/or (*ii*) BFCNs degeneration precedes or paces hippocampal degeneration in the context of DS/AD (Alldred et al., 2023; Alldred et al., 2021c; Mufson et al., 2021).

To evaluate hippocampal neuronal populations, we performed single population gene expression analysis to interrogate CA1 and CA3 pyramidal neurons (PNs) and DGCs. Samples were microisolated by LCM in the same spatial plane, followed by RNA-seq and downstream bioinformatic inquiry to analyze differentially expressed genes (DEGs) and pathway alterations in spatially characterized hippocampal excitatory neuronal populations in the Ts65Dn mice compared to normal disomic (2N) controls at 6 MO. We postulate each excitatory neuron population will display unique gene expression, with a subset of DEGs showing convergent dysregulation in all three populations.

## Materials and methods

### Mice

Ts65Dn (Ts, *n* = 6) and disomic (2N, *n* = 6) male mice (age range: 5.7–6.4 MO, mean age 6.0 MO) were generated as part of previously published studies by our group (Alldred et al., 2023; Alldred et al., 2021c).

### Tissue accession

Brains were accessed as previously described (Alldred et al., 2021c, 2023). Following removal of the brain from the calvarium, a biased hemibrain dissection was performed, isolating one hemisphere ~1–1.5 mm lateral to the midline to preserve centrally located structures, including the basal forebrain nuclei, which was utilized in the previous studies (Alldred et al., 2021c; Alldred et al., 2023). This hemibrain containing the midline structures was flash frozen on dry ice for RNA-seq analysis. Sections of rostral hippocampus were cut on a cryostat (−25°C; CM1860UV, Leica, Buffalo Grove, IL) at a thickness of 20 μm and mounted on polyethylene naphthalate membrane slides (Leica) (Alldred et al., 2024a; Alldred and Ginsberg, 2023; Alldred et al., 2023; Alldred et al., 2021c). Slides were immediately stored under desiccant at-80°C until used for LCM. The other hemisphere (minus the midline structures) from the same mice utilized for RNA-seq was isolated and dissected for a CA1 enriched dissection and a CA3 + DG sector enrichment (Ts, *n* = 6; 2N, *n* = 6). In one 2N case, the other hemisphere was not available, so a 2N littermate was used for protein dissection. Briefly, an ~1.5 mm thick coronal slab was cut from the rostral hippocampus and placed on a dissection microscope (Zeiss Axiosplat). Under magnification (32x), a CA1 sector enriched dissection was isolated from the rest of the hippocampus with the resulting rest of the hippocampus termed CA3 sector + DG enrichment. Each dissected piece was flash frozen on dry ice and stored at-80°C. RNase-free precautions were employed, and solutions were made with 18.2 mega Ohm RNase-free water (Nanopure Diamond, Barnstead, Dubuque, IA).

### Neuron collection

**Figure 1:**
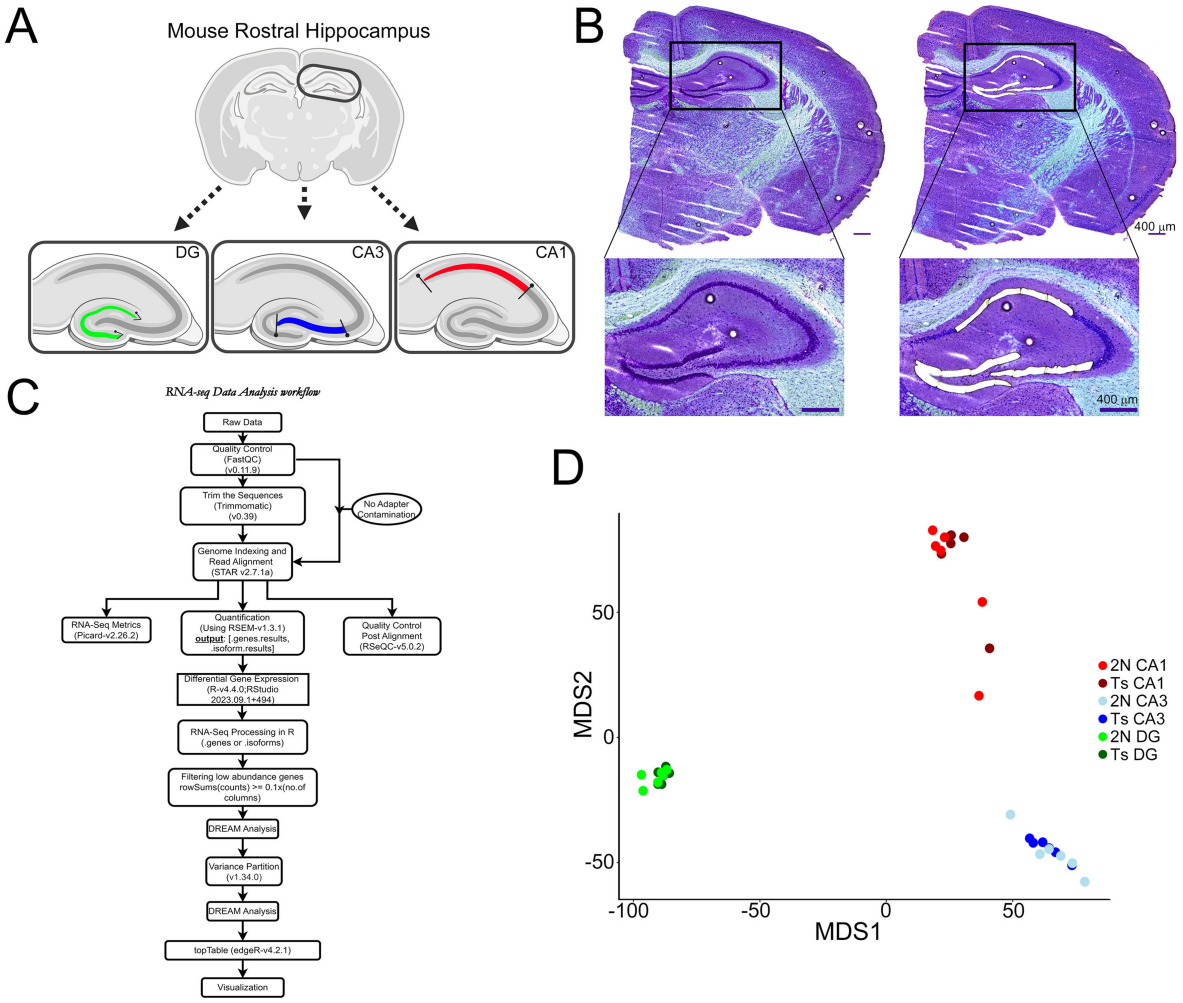
Microisolation and RNA-seq workflow for CA1 PNs, CA3 PNs, and DGCs. **(A)** Schematic representation indicates LCM paradigm to collect excitatory neurons from each hippocampal subregion in a single coronal section (schematic generated via BioRender). **(B)** A representative image shows a coronal section Nissl-stained section at 5x prior to (left) and after (right) microisolation of CA1 PNs, CA3 PNs, and DGCs, with insets showing higher resolution of tightly packed neurons acquired via LCM. Scale bar = 400 μm. **(C)** Overview of the single population RNA-seq bioinformatics analysis paradigm (generated in Drawio). **(D)** MDS plots use a colored dot to represent each individual sample for CA1 PNs (2N = light red; Ts = dark red), CA3 PNs (2N = light blue; Ts = dark blue) and DGCs (2N = light green; Ts = dark green).

Polyethylene naphthalate membrane slides containing the rostral hippocampus were equilibrated to room temperature (RT) under desiccant (−20°C for 5 min, 4°C for 10 min, RT for 5 min) followed by a rapid Nissl staining protocol as previously described to preserve intact RNA in unfixed tissue (Alldred and Ginsberg, 2023). CA1 and CA3 PNs along with DGCs were identified visually in the same section and microisolated using the draw and cut feature by LCM for each section (LMD7000; Leica; Figures 1A,B). As the excitatory neurons are densely packed in these regions, groups of neurons were collected during LCM, with an estimated number of neurons per area collected. Approximately 6 rostral hippocampal tissue sections were dissected via LCM (approximately -1.58 to -2.54 Bregma), with mean collections of ~950 CA1 PNs, ~750 CA3 PNs, and ~ 1,175 DGCs per brain before proceeding to RNA isolation and RNA-seq library preparation.

### RNA purification

RNA from neurons collected for each brain region (CA1, CA3, and DG) was purified using the miRNeasy Micro kit (Qiagen) according to manufacturers’ specifications, which isolates total RNA including microRNAs. A DNase digestion was performed twice sequentially before the final washes and RNA purification. RNA quality control (QC) was performed (High Sensitivity RNA assay, Tapestation, Agilent, Santa Clara, CA). RNA Integrity Number (RIN) values ranged from 5.0–8.2 for all samples quantified, with samples at the limit of resolution measured by DV_200_ values.

### Library preparation and RNA-seq

The SMARTer Stranded Total RNA-Seq kit-Pico input Mammalian v3 (Takara Bio, Mountain View, CA) was employed with unique indexes for each sample. To normalize input, CA1, CA3 and DG RNA concentrations were used to determine sample input, with an average of ~3–3.75 ng RNA input utilized per sample. Samples were fragmented for 3.75 min, with 5 cycles for PCR1 and 14 cycles for PCR2. Samples were quantified (Tapestation D1000 DNA assay; Agilent). One 2N sample for CA1 failed QC for library preparation, so neurons from all three regions (CA1, CA3, and DG) were re-isolated using adjacent tissue sections from the same mouse. LCM, RNA extraction and library preparation were performed and the sample passed QC. Samples were pooled in equimolar concentrations and assayed on a NovaSeq 6000 (Illumina, San Diego, CA) using an S1 100 cycle flow cell (v1.5) by the New York University Grossman School of Medicine Genome Technology Center.

### RNA-seq processing

FastQ files were utilized for both conditions (Ts and 2N) in all three cell types (CA1 PNs, CA3 PNs, DGCs) to analyze data in parallel. FastQ files were generated and QC of the raw reads was performed by FastQC v0.11.9 (Andrews, 2010). Samples were removed from analysis if sequence counts were low (<100 k bases). One CA1 PN Ts sample was removed (<75 aligned reads). Read trimming was then performed as necessary by Trimmomatic 0.39 (Bolger et al., 2014). If QC passed and showed no adapter contamination, this step was skipped. Sequence reads were indexed and aligned to the reference genome (Gencode GRCm39-mm39) using STAR Aligner (2.7.1a) (Dobin et al., 2013). Quantification was performed on alignments using Picard 2.26.2 (Picard Toolkit, 2019) for different measures and RSEM (1.3.1) for output (Li and Dewey, 2011). QC was performed on alignments using RSeQC (v5.0.2) (Wang et al., 2012). Differential gene expression was performed using R version4.4.0/RStudio v1 + 494 using genes results with the mouse reference genome (Gencode GRCm39-mm39) (Figure 1C; Supplementary Figure S1).

### Statistical analysis

Gene Count matrix obtained from RSEM was analyzed. Genes with over 0.1 counts per million for at least 10 samples were retained, TMM normalization was then implemented by edgeR (Robinson et al., 2010) for downstream analysis. This step removes lowly expressed genes as they provide little evidence of differential expression and increase statistical errors and false discovery rate (Zehetmayer et al., 2022; Rau et al., 2013; Robinson et al., 2010). Analyses were performed using the DREAM pipeline (Hoffman and Roussos, 2021) which is built using the limma-voom framework from the VariancePartition (v.1.34.0) package (Hoffman and Schadt, 2016). In addition to Group and RNA concentration, the following variables were included as covariates: Intergenic percentage, Intronic percentage, mRNA base percentage, Usable base percentage, and Correct strand reads percentage. The covariates were computed from RNA-seq reads by Picard, with the exception of Group and RNA concentration. Multidimensional Scaling (MDS) was used to visualize the distribution of points and if necessary, identify the presence of outliers. Rrcov was utilized in R to detect the presence of outliers (Filzmoser and Todorov, 2013; Todorov, 2024). No outliers were detected or removed. TopTable (edgeR; v4.2.1) extracts genes that are present for all comparisons. Gene expression differences at (*p* < 0.05) were considered statistically significant. Protein coding genes were extracted using the R Bioconductor package AnnotationDbi (Pages et al., 2019). Multiple testing corrections were performed by false discovery rate (Broberg, 2005; Figure 1C). To ensure isolated cells were excitatory PNs and GCs, statistical analysis was performed in R using the lmer package to normalized cell counts whereupon excitatory neuronal markers were compared to all other cell specific markers (Mathys et al., 2023) modeled as a function of group (Ts versus 2N) as previously described (Supplementary Figures S2A–C; Alldred et al., 2024b, Alldred et al., 2024a). Significance was judged at the level *α* = 0.05, two-sided.

### Pathway analyses

Pathway analyses consisted of Ingenuity Pathway Analysis (IPA; Qiagen) (Qiagen, 2020; Krämer et al., 2013), Gene Ontology (GO) (Ashburner et al., 2000; Gene Ontology Consortium, 2021) and STRING (Szklarczyk et al., 2018) in Cytoscape (cutoff 0.4) (Shannon et al., 2003). Shiny package (v.1.8.1.1) was utilized to create a web-based app to run GO analysis using R version 4.4.0/ RStudio v1 + 494. This app was also used to filter keyword targets to identify classes of processes affected by genotype and region (Alldred et al., 2024b; Alldred et al., 2024a; Alldred et al., 2023). Overlapping and unique processes were identified using Excel for IPA canonical pathways and neurological diseases and functions (D/Fs), along with GO processes. IPA gene network plots were generated using Igraph (v.2.0.3). STRING analysis was performed (Ts compared to 2N) separately for CA1 PNs, CA3 PNs, and DGCs DEGs to isolate top protein–protein interactions (PPIs) differentially expressed in Ts mice for each cell type. Venn diagrams were generated using InteractiVenn program (Heberle et al., 2015).

### Protein analysis

For protein homogenization, all steps were performed on wet ice or at 4°C. Regional dissections of the hippocampus were utilized to generate the nanogram quantities needed for WES protein analysis (Alldred et al., 2024b; Alldred et al., 2021a; Alldred et al., 2021b). This is technically impractical by LCM (Alldred et al., 2024b; Alldred and Ginsberg, 2023). Each sample received 50 μL of tissue homogenization buffer (THB; 250 mM sucrose, 20 mM Tris base,1 mM EDTA, and 1 mM EGTA) with 1/100 volume of 100 mM phenylmethylsulfonyl fluoride (PMSF; Sigma, P7626) and 1/1000 volume of a protease inhibitor cocktail (Sigma; I3786) as described previously (Alldred et al., 2024b, Alldred et al., 2021a, Alldred et al., 2021b). Manual homogenization using a disposable microfuge pestle was performed for each sample in the microfuge tube, followed by a 5 min centrifugation step at 300 x g to pellet cell debris. The supernatant was extracted to a fresh microfuge tube and stored on wet ice while quantification was performed by the Bradford assay (23236; Coomassie Plus, ThermoFisher, Waltham, MA,) on a Nanodrop 2000C (ThermoFisher). The assay was performed per manufacturer’s specifications with alterations to reduce volume, using bovine serum albumin to generate a standard curve (2 μL protein +58 μL reagent). Once quantification was performed, each sample was diluted to 2 mg/ml concentration using ice-cold THB with protease inhibitors, aliquoted and stored at-20°C until used for protein analysis.

Protein analysis was performed using the WES system (Protein Simple, San Jose, CA). Briefly, 3 μL of each sample was aliquoted to an individual well with 0.8 μL of 5x Fluorescent Master mix following WES protocol guidelines (Alldred et al., 2021a; Alldred et al., 2021b; Alldred et al., 2024b) using the 25 capillary 12–240 kD Wes separation module, to include all 24 samples (*n* = 6/genotype/region) and molecular mass ladder. Blocking reagents, primary and secondary antibodies, chemiluminescent substrate, separation and stacking matrices (Protein Simple) were dispensed to designated wells per manufacturer guidelines. Primary antibodies included mouse anti-*β*-tubulin III (β-TUBIII) used as input control (MAB1195, 1:50, R&D Systems, Minneapolis, MN), amyloid precursor protein (APP; C1/6.1, 1:20, gift of Dr. P.M. Mathews), which recognizes both full length APP as well as the β-CTF fragment (Mathews et al., 2002) and dual specificity tyrosine phosphorylation regulated kinase 1a (DYRK1A; D30C10 #8765, 1:20, Cell Signaling, Danvers, MA). Plates were spun for 5 min at 1000 x g and loaded onto a WES unit, where separation electrophoresis and immunodetection steps are fully automated within the capillary system. Instrument default settings were used with an increased run time to 35 min from default 25 min. Digital images were analyzed with Compass software (Protein Simple), utilizing dropped lines for peak analysis area calculation. Detected proteins were compared to control β-TUBIII levels and reported as normalized percentage of 2N CA1 sector mean. Each protein was performed in triplicate on separate plate runs. Statistical analysis was conducted on each protein compared to β-TUBIII and normalized to 2N CA1 sector mean to standardize means across assay runs. Samples were modeled as a function of the hippocampal region and genotype.

## Results

Herein, we illustrate genotype differences are cell specific in the hippocampus during onset of BFCN degeneration utilizing LCM to isolate three distinct cell types within the hippocampal formation and analyzing gene expression. Using male Ts and 2N mice at the start of BFCN degeneration (~6 MO), CA1 PNs and CA3 PNs, along with DGCs from the rostral hippocampus were identified (Figure 1A) and isolated by LCM (Figure 1B). RNA-seq was performed with subsequent bioinformatic inquiry using the DREAM pipeline in which genotype and cell type differences were identified in Ts versus 2N mice, as seen by voom:mean–variance trend plots using a retention threshold of 10 for all three neuronal populations (Figure 1C; Supplementary Figures S1A–C).

### Hippocampal genotype and cell specific differential gene expression in Ts mice

**Figure 2:**
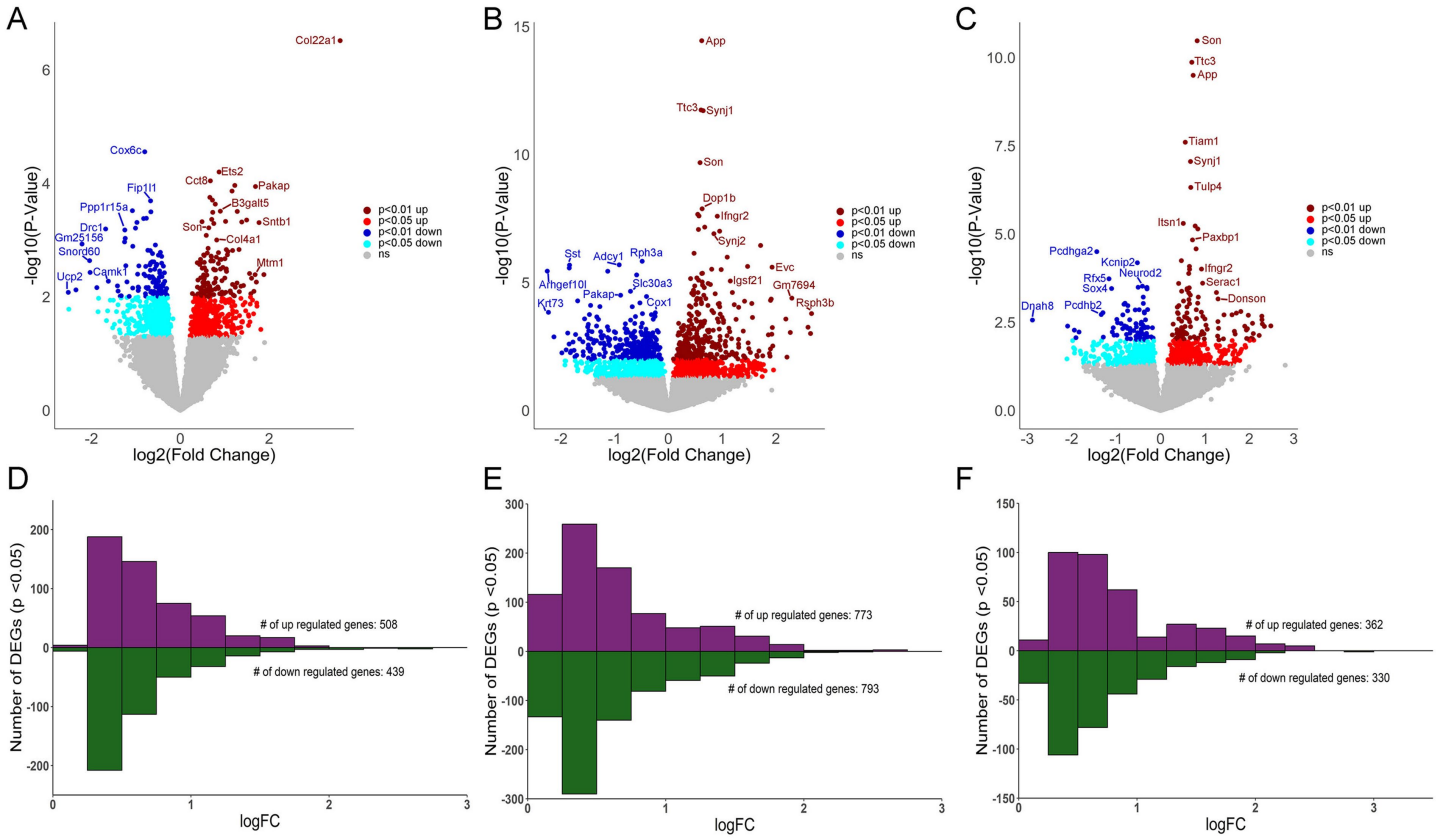
Differential gene expression in CA1 PNs, CA3 PNs and DGCs. **(A)** Volcano plot shows CA1 PN DEGs by genotype with LFC on the *x*-axis and significance [−log (*p*-value)] on the *y*-axis. **(B)** DEGs are both upregulated and downregulated as in a volcano plot in CA3 PNs by genotype. **(C)** Volcano plot depicting DEGs in DGCs by genotype. Key: Dark red dots (*p* < 0.01 upregulated), light red (*p* < 0.05 upregulated), dark blue (*p* < 0.01 downregulated) and light blue (*p* < 0.05 downregulated), with non-significant genes shown in grey. **(D–F)** Bar charts represent upregulated and downregulated DEGs by LFC (binned by 0.25 increments), with the majority of CA1 **(D)**, CA3 **(E)**, and DG **(F)** DEGs displaying <1 LFC difference by genotype (Ts versus 2N).

MDS analysis revealed distinct profiles for CA1 PNs, CA3 PNs, and DGCs (Figure 1D), with less robust differences seen in Ts versus 2N comparisons for all hippocampal neuron populations (Figure 1D; Supplementary Figures S1D–F), paralleling the Uniform Manifold Approximation and Projection (UMAP) analysis demonstrated previously in trisomic mice (Sierra et al., 2024). DEGs (*p* < 0.05) were identified for CA1 PNs, CA3 PNs, and DGCs by genotype (Ts versus 2N), with fewer genotype differences at 6 MO compared to the cell type differences. Interestingly, CA3 PNs had the most DEGs (1,566, Supplementary Table S2), more than CA1 PNs (947 DEGs; Supplementary Table S1) and DGCs (692 DEGs; Supplementary Table S3). Volcano plots show downregulated (*p* < 0.05, light blue; *p* < 0.01, dark blue) and upregulated (*p* < 0.05, red; *p* < 0.01, dark red) DEGs sorted by log-fold change (LFC; base 2) and −log(*p*-value) for CA1 PNs (Figure 2A), CA3 PNs (Figure 2B), and DGCs (Figure 2C). Bar charts were utilized to bin genes by 0.25 increments of the LFC. CA1 PNs (Figure 2D) exhibited more upregulated (508) compared to downregulated (439) DEGs, while CA3 PNs (Figure 2E) and DGCs (Figure 2F) had approximately equal numbers of upregulated and downregulated DEGs. This suggests gene expression defects extend beyond the triplicated region within trisomic hippocampal neurons, with CA3 PNs neurons exhibiting the most robust dysregulation at 6 MO.

**Figure 3:**
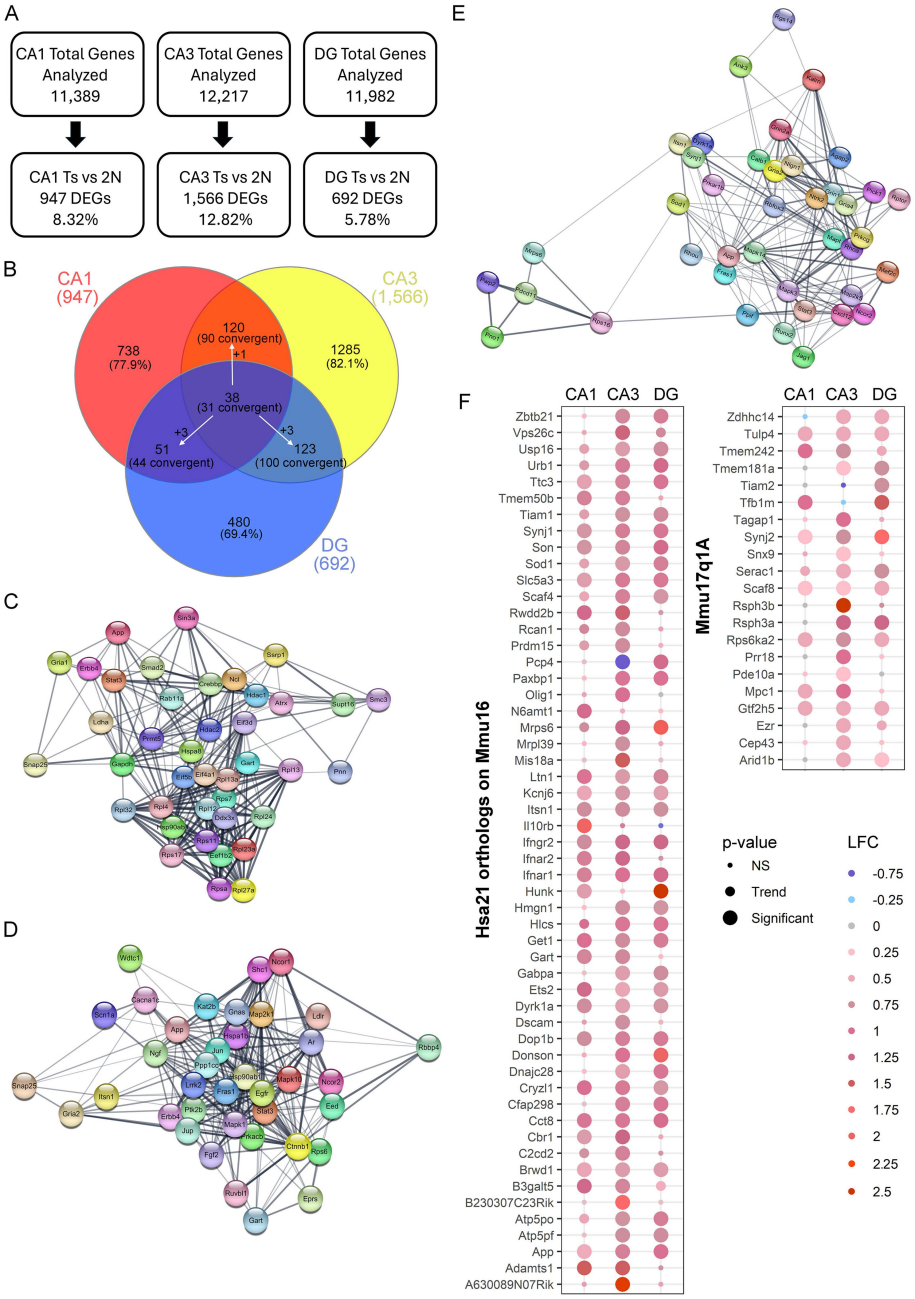
Hippocampal gene expression analysis in excitatory neuron populations. **(A)** Genes passing QC had <10% difference in total expression. Number and/or percentage of DEGs varied by hippocampal neuronal population. **(B)** Venn diagram of DEGs comparing each neuronal population indicated only a few DEGs overlap between CA1 PNs, CA3 PNs, and DGCs. White arrows indicate a DEG was dysregulated uniquely in one population, but convergently dysregulated in the other two. **(C)** STRING plots show the top 40 PPI with at least 29 total PPIs in CA1 PN DEGs, which are presented with each node having a DEG, with edges connecting the interaction partners. **(D)** Plot represents STRING analysis of the top 38 PPI interactors in CA3 PN DEGs which all had at least 41 total PPIs. **(E)** STRING analysis in DGCs showed the top 40 PPIs with overall fewer total PPI partners (>15 partners). STRING plot indicates these PPIs as two unique networks connected by two ribosomal DEGs. **(F)** Dot plots show triplicated DEGs that are HSA21 orthologs (left) or non-orthologous triplicated DEGs from the 17q1a fragment in the Ts mice (right) were identified by *p*-value (size) and LFC (color) with downregulation in shades of blue and upregulation in shades of red.

To ensure the number of DEGs isolated was not caused by overt differences in total number of genes analyzed that passed QC, the percentage of DEGs compared to genes analyzed was identified. While CA3 PNs had the highest total number of genes pass QC, DGCs also had a higher total number pass QC compared to CA1 PNs. However, DGCs had the lowest percentage of DEGs compared to analyzed genes (5.78%), followed by CA1 PNs (8.32%), with CA3 PNs having the highest ratio of DEGs compared to analyzed genes (12.82%; Figure 3A). This suggests that a higher number of genes passing QC may improve statistical analysis of DEGs, but at best, it only partially affects the total DEG outcome.

DEGs for each neuronal population were compared for genotype and circuitry effects, as indicated by convergent and unique gene expression. In each neuronal population, the vast majority of DEGs were unique to each neuronal population (Figure 3B), reinforcing each cell type has a unique expression profile. A total of 38 DEGs were found to be dysregulated in all three excitatory hippocampal populations, with 31 convergently dysregulated. CA1 PNs and CA3 PNs had more overlap of genotype dependent gene expression alterations with 90 total convergent DEGs (Figure 3B). CA1 PNs and DGCs had the least overlap, suggesting genotype dependent changes are highly specialized to cell type, while CA3 PNs and DGCs had the highest number of DEGs expressed in both neuronal populations (Figure 3B). This indicates that CA3 and DG neuronal clusters may have more convergent mechanisms of action which requires evaluation through IPA and GO analysis.

To examine PPIs using DEGs for each neuronal population, STRING in Cytoscape was performed on all DEGs for each region. CA1 PNs had 890 identified protein coding DEGs (of the 947 DEGs), which showed 3,819 total PPIs (Supplementary Figure S3A). These PPIs were then re-examined for the top interactors. STRING was performed on the top 40 PPIs in CA1 PNs with a minimum of 29 PPI partners and indicated a highly interactive PPI network (Figure 3C). CA3 DEGs were examined with 1,482 DEGs identified in STRING, which showed 7,634 total PPIs (Supplementary Figure S3B). The top 38 PPIs with 41 or more PPI partners, showed a closely interacting PPI network, with a highly concentrated cluster of PPI partners at the center of the network (Figure 3D). DGC significant genes (658 protein coding DEGs) showed the fewest interactions (1,705; Supplementary Figure S3C). Interestingly, when the top DEGs were examined in STRING (38 DEGs with 16+ PPI partners), there was a subset of 5 DEGs distinct from the majority of the PPI network (Figure 3E). Only amyloid precursor protein (*App*) is convergently upregulated in all three networks. The paucity of convergent top PPIs in the excitatory neuron hippocampal populations examined suggests changes are likely cell type specific within each hippocampal subregion, with each neuronal population having unique drivers of pathology.

### Triplicated DEGs in spatially characterized hippocampal neurons

To determine whether genes triplicated in trisomic mice drive dysregulation in hippocampal neuron populations, DEGs from each neuronal population were queried. To fully comprehend the effect of the Ts triplicated chromosome, HSA21 orthologs as well as the non-homologous triplicated region (Mmu17q1a; orthologous to HSA6) were analyzed in the Ts mouse model. A significant subset of triplicated genes was both expressed (63 in CA1 PNs, 66 in CA3 PNs, 65 in DGCs: Supplementary Tables S4–S6) and significantly dysregulated in the three hippocampal neuronal populations. Interestingly, CA1 PNs showed the fewest significant triplicated DEGs (27), while CA3 PNs had the most with 51 triplicated DEGs. DGCs had 35 DEGs from the HSA21 triplicated region (Figure 3F, left panel). Only 1 DEG, Purkinje cell protein 4 (*Pcp4*) was significantly downregulated in CA3 PNs, but upregulated in DGCs, while 18 triplicated DEGs were significantly upregulated by genotype in all three neuronal populations (Figure 3F, left panel). To examine dysregulation of the non-disjunctive region (Mmu17q1a) this chromosomal region was queried for DEGs by genotype in the 3 neuronal populations. Upregulation of 6 DEGs in all three hippocampal neuron populations was observed, along with multiple additional non-disjunctive triplicated DEGs upregulated in one or more of the CA1 PN, CA3 PN, and DGC populations (Figure 3F, right panel). Replicating the orthologs, CA1 PNs had the fewest non-orthologous significant DEGs while CA3 PNs had the largest number of upregulated DEGs from Chr17q1A. This suggests triplication in hippocampal neurons may, in part, drive pathology and behavioral changes seen in DS mice, irrespective of whether it is orthologous or disjunctive.

### Mechanistic circuitry dysfunction beyond the DS triplicated region

**Figure 4:**
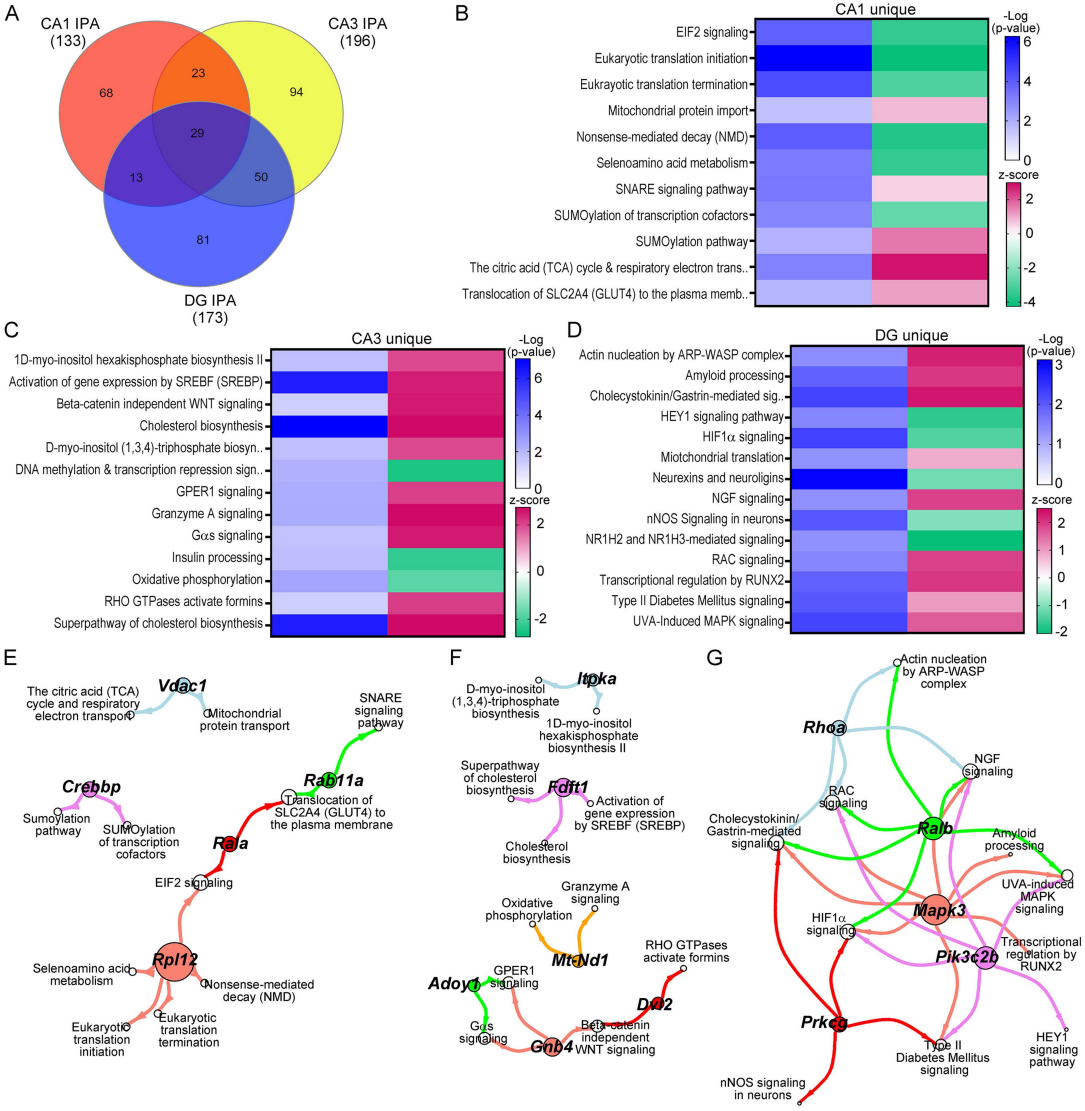
IPA was performed on DEGs for each neuron population. **(A)** Venn diagram indicates a subset of the neuronal pathways overlap between CA1 PNs, CA3 PNs, and DGCs. **(B–D)** Heatmaps of select pathways unique to CA1 PN DEGs **(B)**, CA3 PN DEGs **(C)**, and DGC DEGs **(D)** depict regulation by z-score (upregulated pink, downregulated green), with significance judged using −Log(*p*-value) in shades of blue. **(E–G)** Spiral graphs display top DEGs involved in selected CA1 **(E)**, CA3 **(F)**, and DG **(G)** unique pathways showing little overlap of driver DEGs and select pathways.

While many triplicated gene candidates were dysregulated in these three hippocampal neuronal populations, triplicated DEGs represent a small minority (<5%) of total DEGs for any of the excitatory neuronal populations examined. To determine mechanistic pathways driving functional dysregulation in the Ts mouse model, IPA and GO analysis were performed using DEGs from each hippocampal population. Not surprisingly, IPA analysis mimicked the DEGs themselves, exhibiting uniquely dysregulated pathways for the majority of canonical pathways in CA1 PNs, CA3 PNs, and DGCs in trisomic mice (Figure 4A). Where pathways did overlap between CA1 PNs, CA3 PNs and DGCs, the activity was often divergent, indicating differential mechanisms of action (by z-score; Supplementary Tables S7–S9). Select pathways altered in CA1 PNs included downregulation in nonsense-mediated decay and RNA translation, initiation, and termination, concomitantly with upregulation of SNARE signaling, Tricarboxylic acid and respiratory electron transport, and mitochondrial protein import (Figure 4B). These findings suggest at ~6 MO trisomic CA1 PNs have increased neuronal biogenetic activity. In contrast, CA3 DEGs resulted in upregulation of approximately 80% of the canonical pathways (Supplementary Table S8). Unique pathways included upregulation of cholesterol pathways, beta-catenin independent WNT signaling, and myo-inositol biosynthesis pathways, while downregulated pathways included oxidative phosphorylation, insulin processing and DNA methylation (Figure 4C). These findings suggest CA3 PNs are selectively undergoing oxidative stress and degeneration (Kang et al., 2023; Bayona-Bafaluy et al., 2021; Lind et al., 2020). DGCs, like CA3 PNs, displayed approximately 70% of neuronal pathways were upregulated (Supplementary Table S9). Unique to DGCs, these pathways included neuroprotective pathways such as upregulation of NGF signaling, amyloid processing and mitochondrial translation, with downregulation of nNOS signaling and HIF1α signaling (Figure 4D), suggesting DGCs are exhibiting resilience in trisomic mice.

To determine if specific DEGs were driving multiple pathways, the select CA1 unique pathways were analyzed for driver DEGs. CA1 PNs had 12 driver ribosomal DEGs involved in 5 of the select processes. We highlight ribosomal protein L12 (*Rpl12*) as a representative DEG (Figure 4E). There was little overlap in the remaining pathways, with unique DEGs linking two pathways, with limited overlap between genes and pathways (Figure 4E). CA3 PNs showed a similar outcome. We note each representative DEG underlies 2+ DEGs for each subset of interacting processes (Figure 4F). DGCs exhibited a more centralized pattern of driver DEGs and dysregulated processes, with three driver DEGs involved in six or more of the DG unique pathways, with the two other driver DEGs involved in fewer (4) dysregulated pathways (Figure 4G).

**Figure 5:**
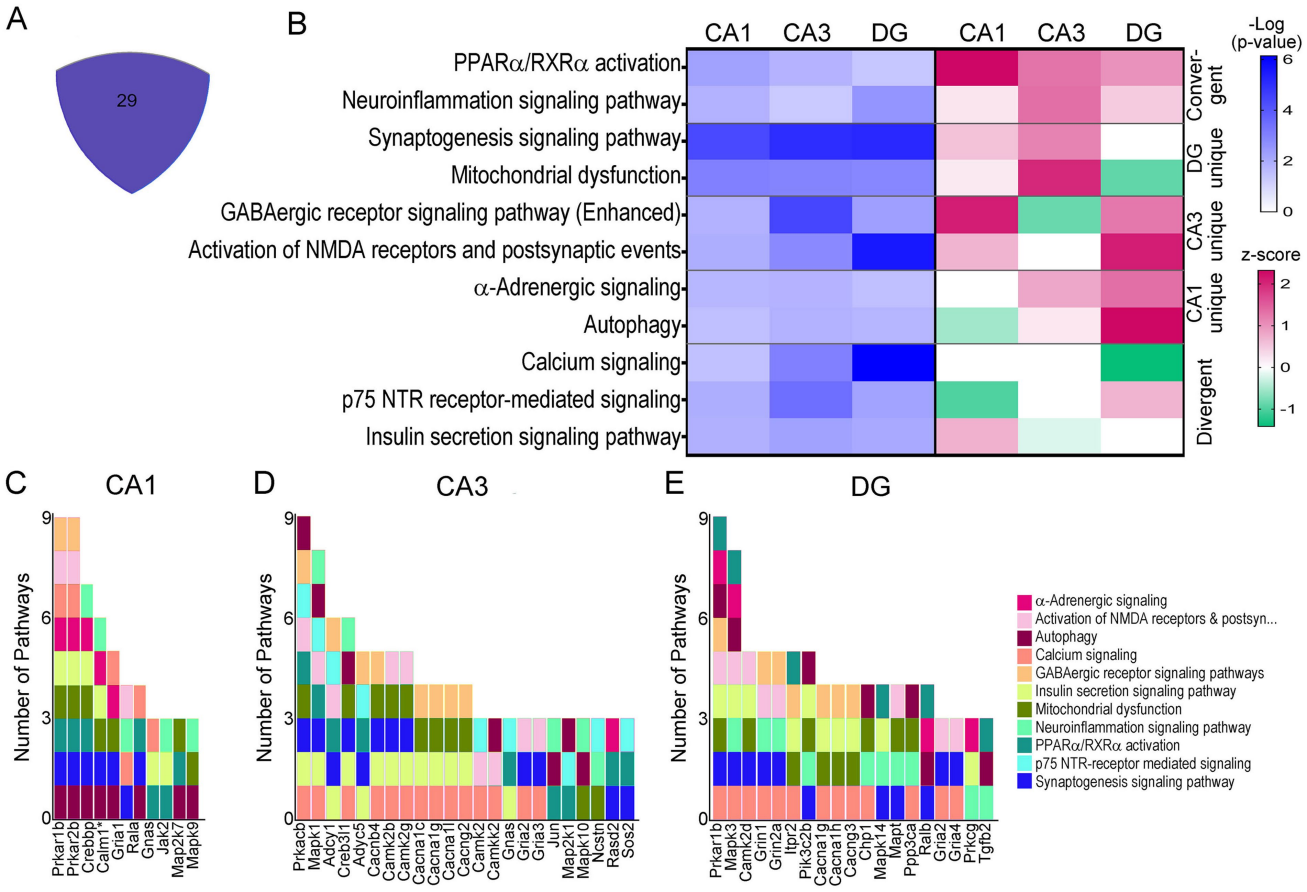
IPA pathways dysregulated in all three neuronal populations. **(A)** Central area of Venn diagram (Figure 4A) highlights the 29 convergently dysregulated pathways. **(B)** In selected overlapping pathways, −Log *p*-value shows significant dysregulation of the convergent pathways by activation score (z-score, pink = upregulation; green = downregulation) **(C–E)** Stacked bar charts show CA1 PN DEGs **(C)**, CA3 PN DEGs **(D)**, and DGC DEGs **(E)** involved in overlapping pathways on the *x*-axis of a bar chart with the *y*-axis linking pathways involved to each DEG. * includes others in CA1 PNs (per the IPA program).

A total of 29 pathways were dysregulated in all three cell types (<25%), when canonical neuronal pathways were interrogated (Figure 5A). To determine activation status, pathways were broken down into convergent (all three upregulated or downregulated by z-score), or divergent, (z-score activation indicating upregulation and downregulation dependent on the cell type). The majority of pathways were divergent in CA1 PNs, CA3 PNs, and DGCs. However, neuroinflammation and PPARα/RXRα activation were convergently upregulated in all three (Figure 5B). Dependent on the pairwise comparison, the divergent overlapping pathways could be considered both convergent and divergent, including mitochondrial dysfunction, which was upregulated in CA1 and CA3 PNs and downregulated in DGCs, GABAergic receptor signaling, which was uniquely downregulated in CA3 PNs while upregulated in CA1 PNs and DGCs, and autophagy which was downregulated in CA1 PNs but upregulated in CA3 PNs and DGCs (Figure 5B).

Pathways indicating unique activation status for all three hippocampal neuronal populations included calcium signaling, which had z-scores of zero for both CA1 and CA3 PNs, indicating there was no clear upregulation or downregulation, but z-scores indicated this pathway was downregulated in DGCs. Similarly, p75^NTR^ receptor signaling showed a z-score indicating downregulation of this pathway in CA1 PNs, no clear activity pattern in CA3 PNs (z-score of 0), and upregulation in DGCs (Figure 5B). To determine whether DEGs themselves reflect why these activation scores are so diverse, driver DEGs were examined. Each neuronal population was examined for drivers of these overlapping pathways (Figure 5B), in many cases this was caused by a unique subunit or isoform dysregulation in trisomic mice. CA1 PNs (Figure 5C), CA3 PNs (Figure 5D), and DGCs (Figure 5E) each display unique genes driving the same dysregulated pathways selected from 29 overlapping pathways. A few DEGs overlapped, including guanine nucleotide binding protein, alpha stimulating (*Gnas*), which was upregulated in CA1 and CA3 PNs, but not significantly in DGCs. *Gria2* is downregulated in CA3 PNs and DGCs but is not significantly in CA1 PNs.

### Neurological Disesae and Functional analysis via IPA reveals molecular phenotypes of unique hippocampal neuron populations

**Figure 6:**
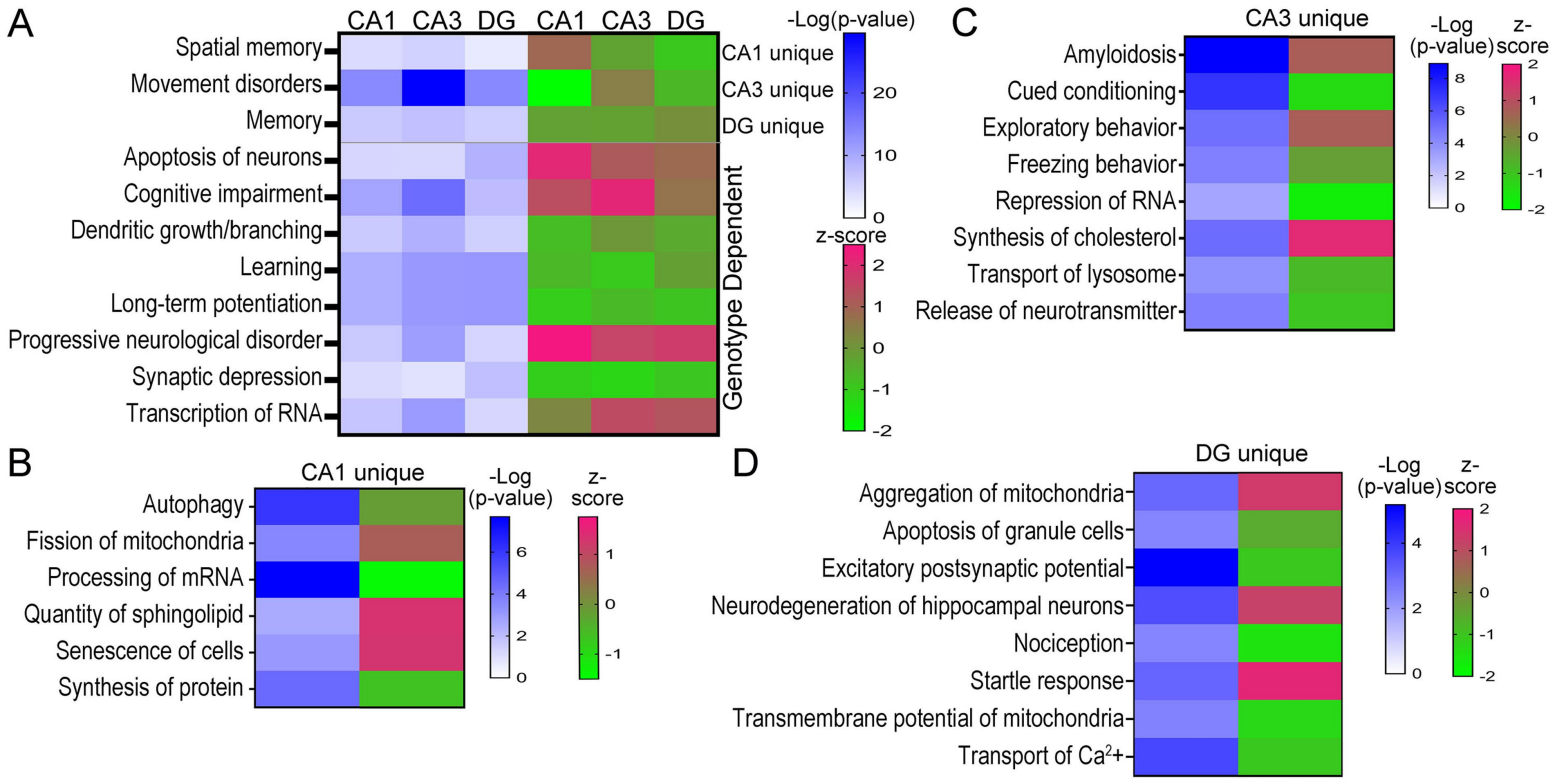
D/Fs in the three hippocampal populations showed neurological deficits in trisomic mice. **(A)** Heatmaps illustrate D/Fs dysregulated in all three neuron populations with significance in shades of blue and activation in shades of pink (upregulated) and green (downregulated). **(B–D)** Select CA1 **(B)**, CA3 **(C)**, and DG **(D)** unique D/Fs are shown by a heatmap indicating significance and activation.

Analysis of D/Fs in IPA was restricted to neurological and cellular D/Fs, which revealed behavioral processes and underlying cellular mechanisms both unique and convergently dysregulated in trisomic hippocampal neuronal populations. Activation z-scores are based on DEGs in the pathway and the reported effect this dysregulation has on the activity of the specific D/F, as determined by the IPA program. Similar to the canonical pathways, many D/Fs were uniquely dysregulated based on the DEG expression, as determined by IPA analysis. Several key behavioral and cellular functions were also dysregulated in all three hippocampal neuron subtypes, including downregulation of learning and LTP and upregulation of progressive neurological disorder (Figure 6A; Supplementary Tables S10–S12). Interestingly, spatial memory was uniquely upregulated in 6 MO trisomic CA1 PNs and downregulated in CA3 PNs and DGCs. Conversely, the D/F termed “memory” was downregulated in CA1 PNs and CA3 PNs and moderately upregulated in DGCs (Figure 6A). Several other D/Fs were only significantly dysregulated in one cell type, including downregulation of autophagy and upregulation of fission of mitochondria in CA1 PNs (Figure 6B; Supplementary Table S10). Trisomic CA3 PNs displayed unique upregulation of amyloidosis and synthesis of cholesterol and downregulation of cued conditioning and repression of RNA (Figure 6C; Supplementary Table S11). DGCs uniquely displayed downregulation of nociception and upregulation of startle response and aggregation of mitochondria (Figure 6D; Supplementary Table S12).

### GO analysis confirms dysregulation is hippocampal cell-type specific

**Figure 7:**
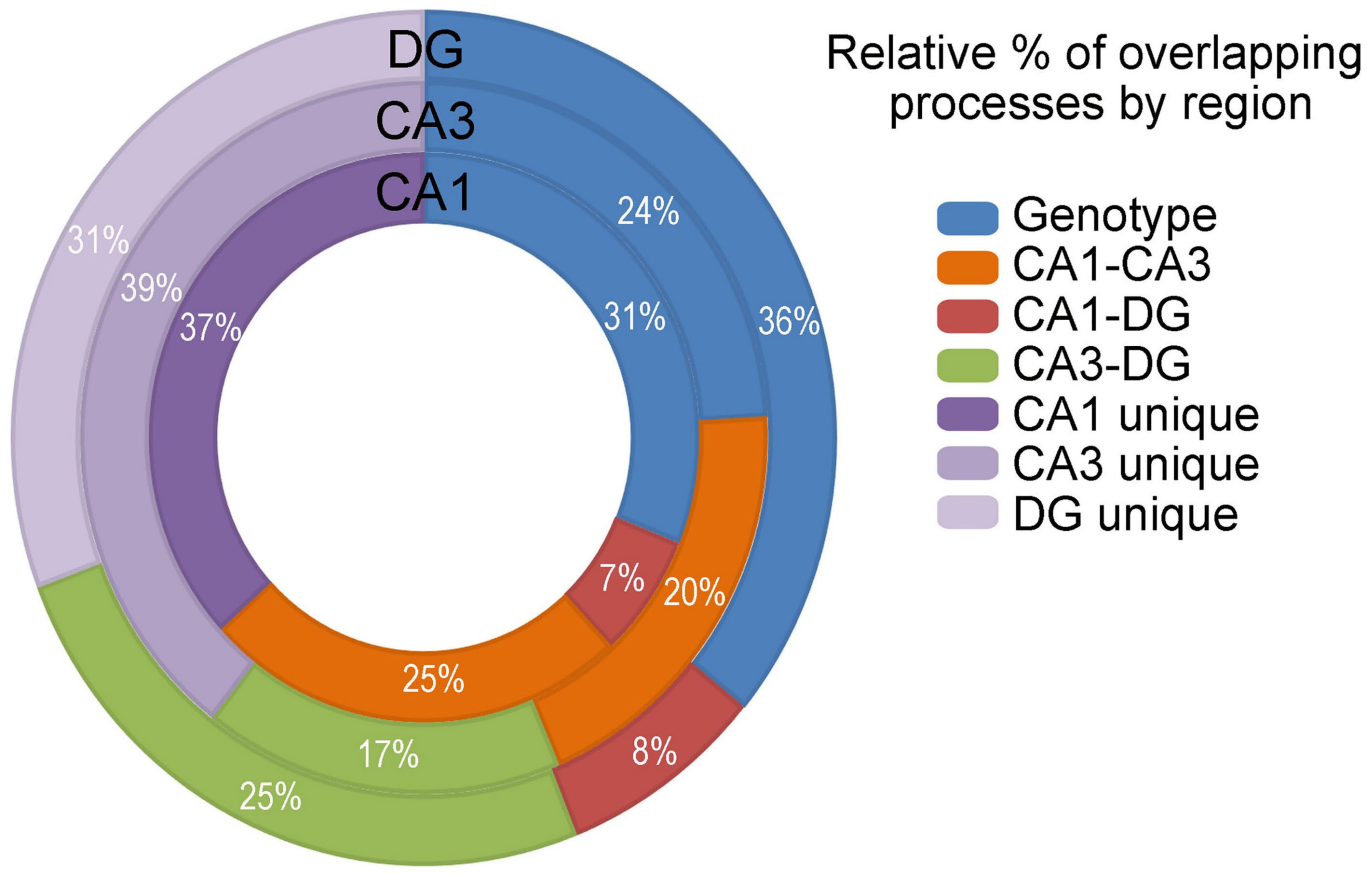
GO analysis was performed and analyzed in the DGCs (outer circle), CA3 PNs (middle circle) and CA1 PNs (inner circle) by genotype (blue), CA1-CA3 overlapping processes (orange), CA1-DG overlapping processes (red), CA3-DG overlapping processes (green), and cell type unique processes (shades of purple). Activation is not accessible in GO analysis. Overlapping processes are assessed and presented.

GO processes, including biological processes, cellular components and molecular functions were analyzed in CA1 PNs, CA3 PNs and DGCs by genotype. Dysfunctional processes were binned into 14 categories (Supplementary Figure S4; Supplementary Tables S13–S15) and analyzed for overlapping or unique processes (Figure 7). CA1 PNs exhibited limited overlap of dysregulated processes with DGCs (7% of total CA1, 8% of total DGCs). In contrast, CA3 PNs showed a higher percentage of overlapping dysregulated processes with DGCs (17% of total CA3 and 25% of total DGCs). DGCs had the most overlapping dysregulated processes (36% of total DGCs), coinciding with the fewest unique dysregulated processes by genotype (31% of total DGCs). Dysregulated trisomic CA1 PNs processes overlapped ~3.5 times more with CA3 PNs than with DGCs. CA3 PNs showed similar percentages of overlap with DGCs or CA1 PNs, although trisomic CA3 PNs had the most unique dysregulated processes. Like IPA, GO analysis indicated a significant minority (31–39%) of dysregulated processes were completely excitatory cell type specific. IPA and GO analysis results in many more pathways and processes driving pathological changes by genotype, while DGC mechanisms appear to be more disparate, suggesting trisomic CA1 and CA3 PNs may drive hippocampal dysregulation concomitantly.

### Protein validation of APP and DYRK1A dysregulation

**Figure 8:**
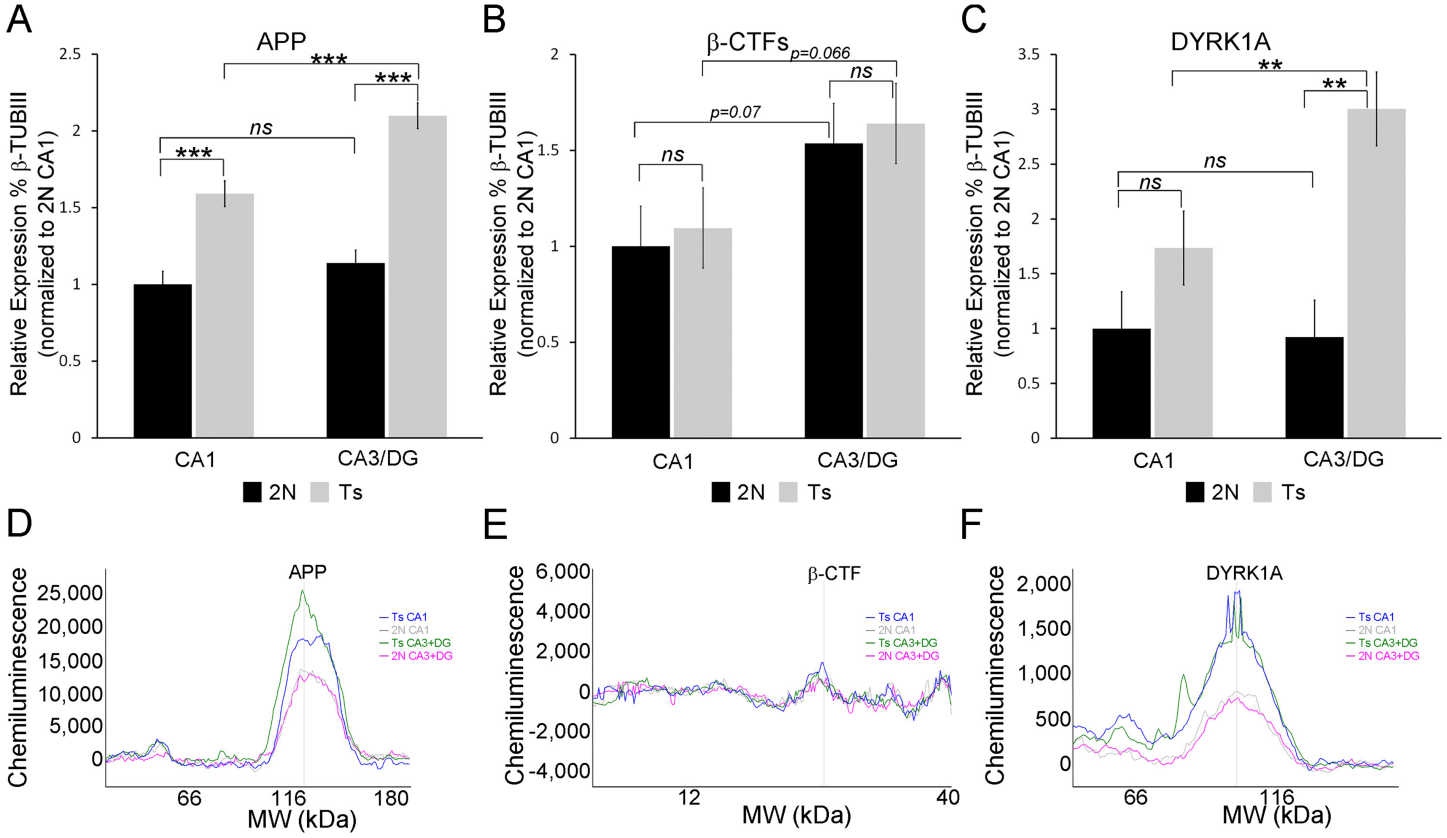
Protein validation was performed on CA1 sector and CA3 sector + DG enriched tissue from the same mice utilized for single population RNA-seq, with the exception of one novel age matched 2N brain as described in the methods **(A)**. APP is upregulated in Ts compared to 2N in both the CA1 sector and CA3 sector + DG enriched dissection. 2N mice showed no difference in expression between regions, while Ts mice showed significant upregulation in CA3 sector + DG compared to CA1. **(B)** β-CTF levels were not significantly different by genotype. A trend level upregulation was found comparing the CA1 sector to CA3 sector + DG enriched dissection. **(C)** DYRK1A expression was not significantly different in the CA1 sector. Significant upregulation was found by genotype in the CA3 sector + DG enriched dissection. Upregulation of DYRK1A expression was also observed in the CA3 sector + DG enriched dissection compared to the CA1 sector in trisomic mice. Proteins were assessed relative to 2N β-TubIII levels and normalized to mean 2N CA1 sector expression. **(D–F)** Representative traces of WES analysis for Ts CA1 (blue), 2N CA1 (grey), Ts CA3 + DG (green), and 2N CA3 + DG (pink) are shown for APP **(D)**, β -CTFs **(E)**, and DYRK1A **(F)**. Key: ***p < 0.001, **p < 0.01, trend levels were presented as *p*-values; ns, not significant.

Two triplicated DEGs were interrogated for protein expression, namely APP and DYRK1A. Both were upregulated in all three hippocampal cell types. *App* displayed varied LFCs with CA1 PNs showing the smallest LFC (Supplementary Table S1, LFC = 0.271), while CA3 PNs and DGCs exhibited larger LFCs (Supplementary Tables S2, S3; LFC of 0.621 and 0.736 respectively). *Dyrk1a*, however, had similar LFCs across all three cell types (LFC range 0.392–0.541; Supplementary Tables S1–S3). At the protein level, APP replicated this pattern with a smaller, but significant, increase in protein expression (~1.59 fold) in CA1 sector enriched tissue (Figure 8A). The CA3 sector + DG enriched dissection also showed significantly increased APP expression by genotype (~1.8-fold, Figure 8A). Regional differences were not seen in APP expression for 2N mice. However, trisomic mice showed significant regional upregulation in the CA3 sector + DG dissection versus the CA1 sector (Figure 8A). *β*-CTF fragments did not reach statistical significance in either the CA1 sector or CA3 sector + DG dissection (Figure 8B). Similar to APP, the CA3 sector + DG dissection displayed a trend for higher expression of β-CTF compared to the CA1 sector in both 2N (*p* = 0.072) and Ts (*p* = 0.066; Figure 8). DYRK1A showed high variability in the CA1 sector, resulting in no significant difference in protein expression by genotype. Significant upregulation of DYRK1A was observed in the CA3 sector + DG dissection in trisomic mice (Figure 8). No significant differences were seen between the CA1 sector and the CA3 sector + DG dissection in 2N mice. Similar to APP protein expression, DYRK1A exhibited lower expression in the CA1 sector compared to the CA3 sector + DG dissection in trisomic mice (Figure 8, *p* < 0.0031). The protein assays were conducted in subregional dissections containing admixed cell types. Results show elevated APP expression suggesting expression increases in both neuronal and non-neuronal (e.g., astrocyte and/or microglia) populations which has been previously seen after neuronal damage (Zhao et al., 2011). However, DYRK1A protein expression increases are partially masked, which may suggest DYRK1A expression is neuron specific, as previously described in postmortem human tissue (Wegiel et al., 2004). Overall, triplicated protein expression appears to mimic alterations in RNA expression in the trisomic model.

## Discussion

RNA-seq analysis of Ts65Dn mice in three hippocampal excitatory neuronal populations resulted in unique differences in gene expression in CA1 PNs, CA3 PNs, and DGCs. CA3 PNs showed the most profound dysregulation, with more DEGs (Figure 2; Supplementary Table S2), resulting in higher numbers of dysregulated canonical pathways and processes by genotype (Supplementary Tables S8, S11, S14). Transcriptomic data corroborates previous studies showing dysregulation of CA3 mEPSCs in the Ts65Dn mouse model (Hanson et al., 2007; Stagni et al., 2013), with transcriptomic analysis indicating specific upregulation of NMDA receptors and downregulation of GABAergic signaling (Figure 5B). However, to date no studies replicated this excitatory effect in CA1 PNs or DGCs. Upregulation of HSA21 orthologs as well as non-homologous triplication of Mmu17 genes were seen in all three neuronal populations, with unique as well as convergent dysregulation of a subset of the triplicated region (Figure 3F). Thus, our in-depth analysis of CA1 PNs, CA3 PNs, and DGCs by genotype resulted in key transcriptomic differences associated with signaling and circuitry alterations in this DS/AD model.

To determine the effect of triplication on gene expression throughout the hippocampal excitatory neuron network, DEGs for each neuronal type were examined for HSA21 orthologs. Increased expression in one or more neuronal populations was seen for a significant subset of the triplicated orthologs, as seen previously (Sierra et al., 2024). Interestingly, half of the 31 convergent DEGs in the three hippocampal cell types (Figure 3B) were HSA21 orthologs (Figure 3F). This included *App* and *Dyrk1a,* which were further examined for protein expression. Previous studies using trisomic mouse hemibrains found disomic APP levels were maintained until ~8 MO (Choi et al., 2009), while microarray analysis revealed upregulation of *App* CA1 PNs in older (10+ MO) but not 6 MO Ts65Dn mice (Alldred et al., 2015b; Alldred et al., 2015a). These prior studies suggest hippocampal APP protein levels are increased at an earlier timepoint than the rest of the brain. Although these findings were not observed by microarray analysis (Alldred et al., 2015a), the higher sensitivity of RNA-seq based assays allows for the statistically significant identification of small increases, as seen in a recent single nucleus RNA-seq analysis (Sierra et al., 2024). However, clinical trials with treatments (e.g., immunotherapies) targeting Aβ have equivocal results in regard to cognitive benefits in AD dementia (Solopova et al., 2023; Ebell et al., 2024; Digma et al., 2024). We postulate the mechanisms driving DS/AD pathology result from additional factors beyond APP triplication (Mufson et al., 2021; Alldred et al., 2024b; Alldred et al., 2024a). In this regard, intellectual disabilities, linked to hippocampal *Dyrk1a* overexpression, are ameliorated when *Dyrk1a* expression is reduced in DS mouse models (Altafaj et al., 2013; Dowjat et al., 2007; Feki and Hibaoui, 2018). Significant DYRK1A encoded protein upregulation was seen in the CA3 sector + DG tissue, along with increased RNA expression seen in all three hippocampal neuronal populations. We postulate the DYRK1A protein is upregulated in neurons throughout the hippocampus and is causal to memory impairments. Support comes from studies that show adult human neurons exhibit higher expression levels of DYRK1A compared to relatively low expression levels in glial populations in normal postmortem brain tissue (Wegiel et al., 2004). Moreover, DYRK1A expression is upregulated within excitatory cortical PNs of individuals with DS (Alldred et al., 2024b).

We noted 5 of the 31 convergent DEGs were HSA6 orthologs, homologous to the non-disjunctive triplicated region of Mmu17, fragment 1A (Chr17q1A; Figure 3F; Duchon et al., 2011), indicating off target upregulation is also seen in these hippocampal neurons. However, while a large subset of triplicated DEGs were identified from each neuronal subtype, accounting for the majority of convergent DEGs overall, these DEGs represent a minor fraction of the total dysregulation observed in trisomic neurons, thus we concentrated on studying gene expression beyond triplicated HSA21 orthologs.

To examine genotype and circuitry effects on cellular mechanisms, DEGs were examined using IPA and GO analysis. Many pathways and processes overlapped between neuronal populations. However, unique gene expression changes often underlie these dysregulated pathways in trisomic mice, with alternative subunits or isoforms dysregulated in each neuronal population. For example, the top genes dysregulated in 9 convergent IPA pathways in trisomic CA1 PNs were protein kinase cAMP-dependent type I regulatory subunit beta (*Prkar1b*; Figure 5C) and protein kinase cAMP-dependent type II regulatory subunit beta (*Prkar2b*; Figure 5C), while in the CA3 PNs, the top gene dysregulated in those same 9 pathways was protein kinase cAMP-activated catalytic subunit beta (*Prkacb*; Figure 5D). Subunit specificity of the protein kinase A (PKA) holoenzyme modulates subcellular targeting and PKA functional specificity (Omar and Scott, 2020). This suggests secondary regulation is highly specialized in each trisomic hippocampal neuron population, each with their own intrinsic vulnerabilities. This example is one of many DEGs displaying subunit specificity for common pathways dysregulated based on the spatial localization and circuitry differences from excitatory hippocampal neurons. We postulate dysregulation of cellular mechanisms is dependent on innervation and circuitry activation in the hippocampus, especially from the perforant path and BFCNs, driving unique behavioral alterations and neurological functions (Mufson et al., 2016; Ginsberg, 2010; Ginsberg et al., 2006; Alldred et al., 2024a; Alldred et al., 2023; Alldred et al., 2021c).

IPA analysis revealed many D/Fs were population specific. CA1 PNs showed upregulation of spatial memory, while CA3 PNs and DGCs DEGs resulted in downregulation of this pathway by genotype (Figure 6A), indicating gene expression alterations are compensatory for spatial memory functions within CA1 PNs. Although CA1 PNs are thought to drive spatial memory and consolidation of spatial memory as part of the septohippocampal circuit (Ash et al., 2014; Velazquez et al., 2013; Al-Onaizi et al., 2017; Perez-Cruz et al., 2011), increased cholinergic tone in Ts65Dn mice by ~7–8 MO has also been suggested to be a compensatory mechanism for the progressive increase in dysfunction similar to prodromal AD (Kelley et al., 2016). BFCN innervation to the CA1 sector may preserve or compensate for degeneration at this younger age. Together, we postulate connectivity-based alterations in the septohippocampal circuit drive memory, specifically spatial memory alterations, associated with CA1 neurons, which is preserved early, but ultimately is lost during the progression of BFCN degeneration in trisomic mice (Alldred et al., 2021c).

We link unique pathways and processes significantly dysregulated in CA1 PNs, CA3 PNs, and DGCs to previous behavioral assessments. IPA predicts DEGs in CA3 PNs will drive increased exploratory behavior, which coincides with increased amyloidosis (Figure 6), suggesting CA3 PNs are degenerating, as trisomic mice have previously been shown to have impaired fear conditioning by 4–6 MO (Costa et al., 2008). Fear conditioning is partially rescued by the NMDA receptor antagonist, memantine, by increasing activity of excitatory neurons (Costa et al., 2008). This corroborates the link between impaired excitatory CA3 PNs and behavioral deficits previously seen in trisomic mice. IPA and GO analyses reveal CA3 PNs have a robust degenerative phenotype, with DEGs and memory-related processes downregulated, suggesting CA3 PNs are significantly impaired at this early timepoint. DGCs display decreases in excitatory postsynaptic potential (Figure 7D). Previous studies suggest this decrease is due to overinhibition driven by inhibitory neuron signaling within the DG (Kleschevnikov et al., 2004; Kleschevnikov et al., 2012b). However, DEGs identified in the D/F “Excitatory postsynaptic potential” were heavily linked to dsyregulation of AMPA/NMDA receptor subunits (Supplementary Table S12), as expected when exclusively profiling excitatory neuron populations. Further, trisomic mice exhibited genotype dependent decreased LTP (Figure 6A) in all three excitatory neuron populations. Decreased LTP has been associated with an excitatory/inhibitory imbalance (Kleschevnikov et al., 2012b; Kleschevnikov et al., 2004; Costa and Grybko, 2005; Chakrabarti et al., 2010). We posit that both excitatory and inhibitory neurons have dysfunctional gene expression changes that cumulate in mechanistic alterations of cellular functions, which underlie degeneration in this well-established DS/AD model. Further study of trisomic inhibitory neurons is required to corroborate these findings.

Molecular hallmarks of the DS degenerative phenotype are seen in Ts65Dn mice, with profound deficits within MSN BFCNs (Alldred et al., 2023) as well as a mosaic of deficits in the three populations of hippocampal excitatory neurons. When examining septohippocampal circuit neurons, hippocampal excitatory neurons show many more triplicated DEGs compared to MSN BFCNs (Alldred et al., 2023), suggesting genotype effects from triplication are more profound within hippocampal excitatory neurons compared to BFCNs. In contrast, total DEGs and the underlying mechanistic pathways dysregulated in Ts65Dn mice are more profound in MSN BFCNs (Alldred et al., 2023), compared to the hippocampal neuron populations. Early deficits, including increased neuroinflammation and reactive oxygen species generation, are seen in both MSN BFCNs and hippocampal neurons by 6 MO, along with synaptic and metabolic deficits, which are much more profound in BFCNs (Alldred et al., 2023), suggesting BFCN dysregulation precedes or paces hippocampal neurons deficits via connectivity-based neurodegeneration. This is corroborated by previous studies in human AD and DS (Yates et al., 1980; Whitehouse et al., 1982) and DS mice (Granholm et al., 2000; Kelley et al., 2014a; Lockrow et al., 2009), which display profuse age-dependent BFCN degeneration. Further, degeneration of the basal forebrain occurs prior to the entorhinal cortex in AD (Fernández-Cabello et al., 2020). We postulate that early targeting of BFCNs likely would slow or stop the onset of hippocampal degeneration.

### Caveats and future directions

We strive to limit LCM and RNA variability by normalizing quality and quantity during bioinformatic analysis. Sex differences exist in AD pathology and this study lacks female animals, which results in the inability to determine sex effects. We plan to ameliorate this deficit utilizing a female cohort, age-and sex matched to the males performed herein, although sex effects have not been noted in human DS RNA-seq in cortical excitatory neurons (Alldred et al., 2024b). To isolate genotype specific differences driven solely by BFCN degeneration, it would be advisable to utilize a younger cohort prior to frank neurodegeneration. Further, new models of DS (e.g., TcMAC21 mouse) (Kazuki et al., 2020; Shao et al., 2023) have been developed that reproduce triplication of virtually all HSA21 orthologs, which may result in unique DEGs not seen herein. Additional planned assessments include examining the therapeutic modality of maternal choline supplementation in hippocampal cell types, as trisomic BFCNs display notable benefits and reduction of dysregulated DEGs and pathways in the Ts65Dn model (Alldred et al., 2023). We also propose examining GABAergic interneurons in the septohippocampal circuit for deficits in the context of DS/AD.

## Conclusion

Using single population profiling, we analyzed alterations in an established DS/AD model at the onset of septohippocampal degeneration and identified genotype and circuitry specific alterations. As expected, each excitatory population had a unique expression profile, but unexpectedly, few genes exhibited convergent dysregulation in excitatory neurons throughout the hippocampus in trisomic mice. We postulate unique gene expression within each circuit drives pathology in the DS brain. Interestingly, CA3 PNs exhibited the most robust dysregulated gene expression profile, while CA1 PNs and DGCs exhibited fewer dysregulated DEGs. Overall, bioinformatic analysis indicated significant overlap in pathway dysregulation were associated with unique DEGs, suggesting isoform and/or subunit specificity is linked to circuitry and/or expression to individual hippocampal neuronal populations, which has translational implications for human DS and informs on AD dementia. Bioinformatic inquiry of CA1 PN, CA3 PN, and DGC DEGs delineated unique drivers of disease pathology and linked select behavioral deficits to individual excitatory neuronal populations dysregulated in trisomic mice. We propose unique gene expression changes may drive similar outcomes in different neuronal populations through distinct regulation of signaling cascades, which in turn suggests precision targeting for therapeutic modulation of degeneration may be necessary for DS/AD degeneration.

## Data Availability

The datasets presented in this study can be found in online repositories. The names of the repository/repositories and accession number(s) can be found in the article/Supplementary material. RNA-seq data analyzed within this study are available from GEO (http://www.ncbi.nlm.nih.gov/geo; GSE283699).

## Supplementary material

SUPPLEMENTARY FIGURE S1 **(A–C)** Gene expression analysis using voom showed mean-variance trends at a stringency of 10 for isolation of DEGs by genotype in CA1 PNs **(A)**, CA3 PNs **(B)**, and DGCs **(C)**. **(D)** MDS plots for CA1 PNs show inter-and intra-group variability for 2N (light red) and Ts (dark red) per subject. **(E)** CA3 PNs MDS with 2N (light blue) and Ts (dark blue) dots per subject. **(F)** DGCs MDS plot shows 2N (light green) and Ts (dark green) variability per subject.

SUPPLEMENTARY FIGURE S2 Normalized cell counts were assayed to determine gene expression for markers of eight different cell types (Mathys et al., 2023). **(A–C)** LCM microisolated hippocampal neurons showed significantly higher expression for excitatory neuron (ExcN) expressing genes compared to genes identified as expressing specifically in inhibitory neurons (InhN), astrocytes (Astro), oligodendrocytes (oligo), oligodendrocyte progenitor cells (OPC), microglia (MG), endothelial cells (Endo), and T-cells (T). CA1 **(A)** *p* < 0.0001 for all comparisons. No significant difference was seen between ExcN expression by genotype (*p* = 0.4382). CA3 PNs **(B)** 2N *p* < 0.0001 for all comparisons; Ts *p* < 0.0011 or lower for all comparisons. No significant difference was seen between ExcN expression by genotype (*p* = 0.2594). **(C)** DGCs *p* < 0.0001 for all comparisons. No significant difference was seen between ExcN expression by genotype (*p* = 0.7071).

SUPPLEMENTARY FIGURE S3 STRING in Cytoscape analysis was performed on all DEGs by cell type and genotype. **(A)** Of the 948 DEGs, STRING identified 890 proteins to examine for PPI analysis. Clusters show significant overlap of PPI s of CA1 PNs DEGs, with few DEGs showing none or only 1 interacting partner. **(B)** Of the 1,566 DEGs in CA3 PNs, STRING found 1,482 proteins for PPI analysis. Clusters show significant overlap of PPI interactions of CA1 DEGs, with CA3 DEGs showing the lowest number of proteins with 1 or no interacting partners. **(C)** Of the 692 DGCs DEGs, 658 proteins were queried for PPI analysis. DGCs had the highest total number of proteins showing no or one interaction with the other proteins queried from the DEGs.

SUPPLEMENTARY FIGURE S4 GO analysis was employed to interrogate DEGs from the three hippocampal neuron populations. **(A)** Processes dysregulated in GO analysis in CA1 PNs were binned by category, with “protein” having relatively more dysregulation (percentage of total dysregulated pathways) compared to CA3 PNs or DGCs. **(B)** CA3 PNs showed similar dysregulation in terms of percentages of processes compared to CA1 PNs, with only slightly higher rates of metabolism and neurotransmitter, ions and receptor (NIR) processes dysregulated, although NIR had a higher percentage in DGCs compared to CA3 PNs. **(C)** DGCs showed relatively more dysregulated developmental processes, more than CA1 or CA3 and a moderately higher relative percentage of signaling processes were dysregulated. Signaling percentage was lowest in CA1 PNs, moderate in CA3 PNs, and highest in DGCs.

SUPPLEMENTARY TABLE S1 List of DEGs for CA1 PNs in Ts compared to 2N at *p* < 0.05.

SUPPLEMENTARY TABLE S2 List of DEGs for CA3 PNs in Ts compared to 2N at *p* < 0.05.

SUPPLEMENTARY TABLE S3 List of DEGs for DGCs in Ts compared to 2N at *p* < 0.05.

SUPPLEMENTARY TABLE S4 List of all triplicated genes expressed in CA1 PNs including orthologs and non-orthologs.

SUPPLEMENTARY TABLE S5 List of all triplicated genes expressed in CA3 PNs including orthologs and non-orthologs.

SUPPLEMENTARY TABLE S6 List of all triplicated genes expressed in DGCs including orthologs and non-orthologs.

SUPPLEMENTARY TABLE S7 List of IPA canonical pathways significantly dysregulated in CA1 PNs in Ts compared to 2N.

SUPPLEMENTARY TABLE S8 List of IPA canonical pathways significantly dysregulated in CA3 PNs in Ts compared to 2N.

SUPPLEMENTARY TABLE S9 List of IPA canonical pathways significantly dysregulated in DGCs in Ts compared to 2N.

SUPPLEMENTARY TABLE S10 List of IPA D/Fs significantly dysregulated in CA1 PNs in Ts compared to 2N.

SUPPLEMENTARY TABLE S11 List of IPA D/Fs significantly dysregulated in CA3 PNs in Ts compared to 2N.

SUPPLEMENTARY TABLE S12 List of IPA D/Fs significantly dysregulated in DGCs in Ts compared to 2N.

SUPPLEMENTARY TABLE S13 Total GO processes sorted by bin of significantly dysregulated in CA1 PNs in Ts compared to 2N.

SUPPLEMENTARY TABLE S14 Total GO processes sorted by bin of significantly dysregulated in CA3 PNs in Ts compared to 2N.

SUPPLEMENTARY TABLE S15 Total GO processes sorted by bin of significantly dysregulated in DGCs in Ts compared to 2N.

## Glossary

2N: Normal disomic controls
A*β*: Amyloid-beta peptide
AD: Alzheimer’s disease
APP: Amyloid precursor protein
*β*-TUBIII: β-tubulin III
BFCNs: Basal forebrain cholinergic neurons
DEGs: Differentially expressed genes
D/Fs: Disease and functions
DGCs: Dentate gyrus granule cells
DS: Down syndrome
DYRK1A: Dual specificity tyrosine phosphorylation regulated kinase 1a.
*Gnas*: Guanine nucleotide binding protein, alpha stimulating
GO: Gene Ontology
HSA21: Human chromosome 21
IPA: Ingenuity Pathway Analysis
LCM: Laser capture microdissection
LFC: Log-fold change
LTP: Long-term potentiation
MDS: Multidimensional Scaling
Mmu16: Mouse chromosome 16
Mmu17: Mouse chromosome 17
MO: Months of age
*Pcp4*: Purkinje cell protein 4
PN: Pyramidal neuron
PPI: Protein–protein interaction
*Prkar1b*: Protein kinase cAMP-dependent type I regulatory subunit beta
*Prkar2b*: Protein kinase cAMP-dependent type II regulatory subunit beta
QC: Quality control
RIN: RNA Integrity Number
RNA-seq: RNA-sequencing
*Rpl12*: Ribosomal protein L12
RT: Room temperature
THB: Tissue homogenization buffer
UMAP: UNIFORM Manifold Approximation and Projection
