# Supplementary figures and images for: Profiling hippocampal neuronal populations reveals unique gene expression mosaics reflective of connectivity-based degeneration in the Ts65Dn mouse model of Down syndrome and Alzheimer’s disease
Source: Front Mol Neurosci. 2025 Feb 26;18:1546375. doi: 10.3389/fnmol.2025.1546375 (PMC11897496; doi:10.3389/fnmol.2025.1546375)

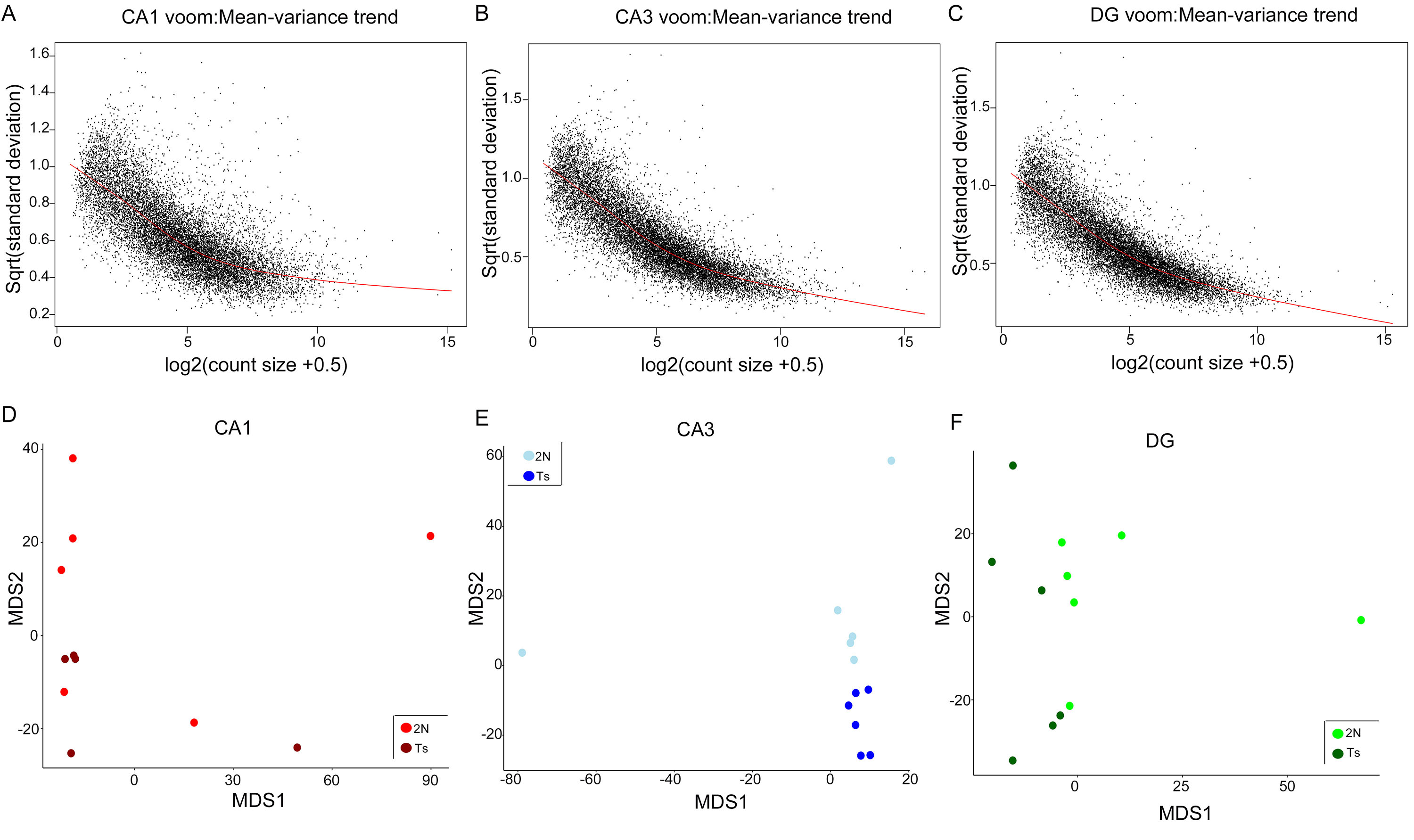

Supplement: SUPPLEMENTARY FIGURE S1 — (A–C) Gene expression analysis using voom showed mean-variance trends at a stringency of 10 for isolation of DEGs by genotype in CA1 PNs (A), CA3 PNs (B), and DGCs (C). (D) MDS plots for CA1 PNs show inter-and intra-group variability for 2N (light red) and Ts (dark red) per subject. (E) CA3 PNs MDS with 2N (light blue) and Ts (dark blue) dots per subject. (F) DGCs MDS plot shows 2N (light green) and Ts (dark green) variability per subject. [file Image_1.JPEG]

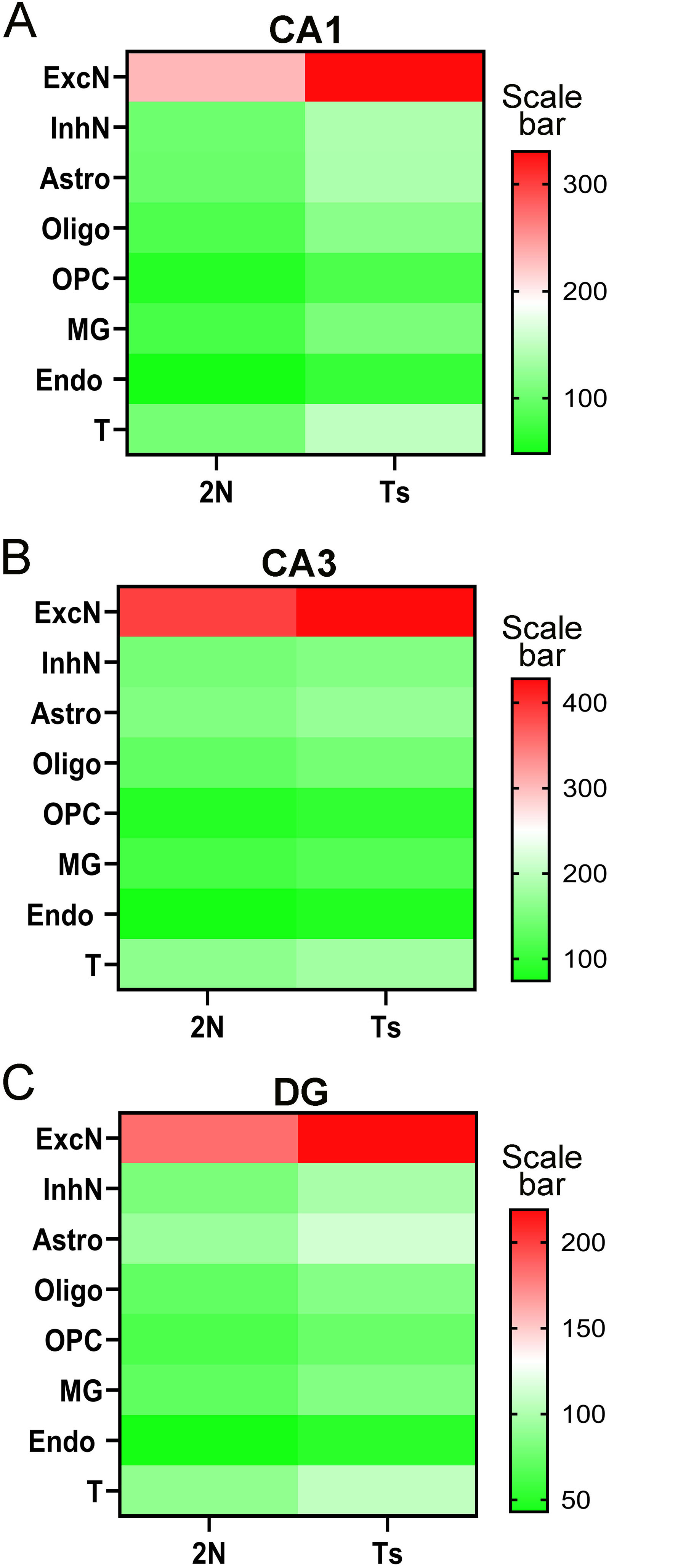

Supplement: SUPPLEMENTARY FIGURE S2 — Normalized cell counts were assayed to determine gene expression for markers of eight different cell types (Mathys et al., 2023). (A–C) LCM microisolated hippocampal neurons showed significantly higher expression for excitatory neuron (ExcN) expressing genes compared to genes identified as expressing specifically in inhibitory neurons (InhN), astrocytes (Astro), oligodendrocytes (oligo), oligodendrocyte progenitor cells (OPC), microglia (MG), endothelial cells (Endo), and T-cells (T). CA1 (A) p < 0.0001 for all comparisons. No significant difference was seen between ExcN expression by genotype (p = 0.4382). CA3 PNs (B) 2N p < 0.0001 for all comparisons; Ts p < 0.0011 or lower for all comparisons. No significant difference was seen between ExcN expression by genotype (p = 0.2594). (C) DGCs p < 0.0001 for all comparisons. No significant difference was seen between ExcN expression by genotype (p = 0.7071). [file Image_2.JPEG]

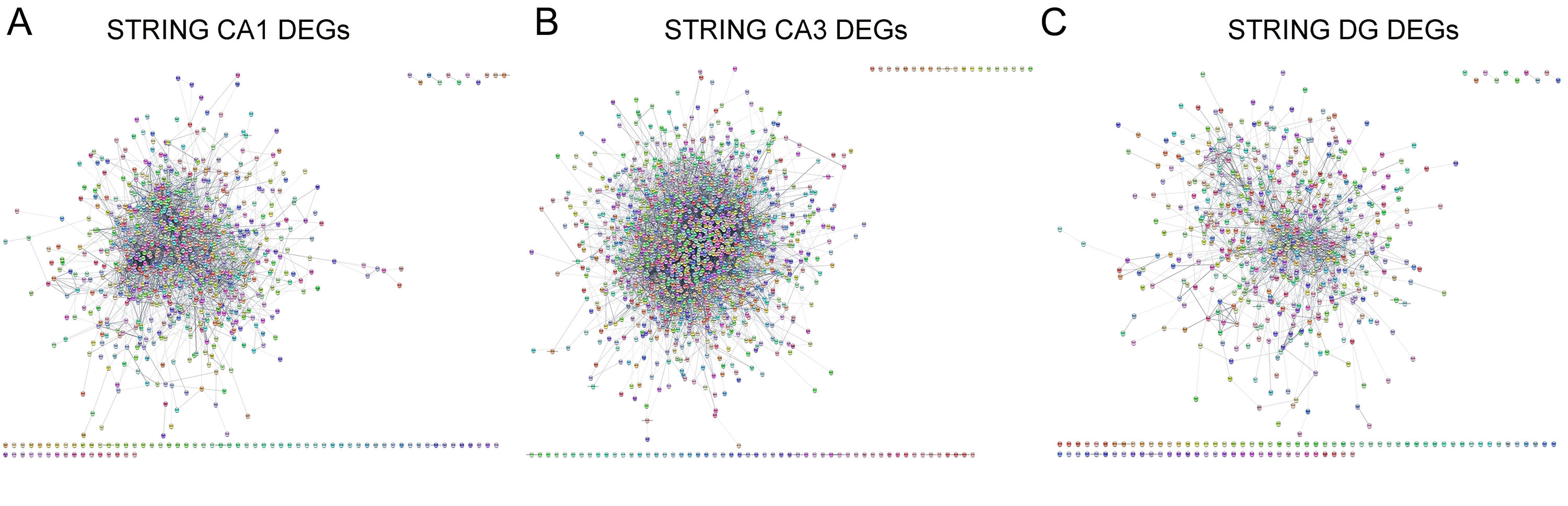

Supplement: SUPPLEMENTARY FIGURE S3 — STRING in Cytoscape analysis was performed on all DEGs by cell type and genotype. (A) Of the 948 DEGs, STRING identified 890 proteins to examine for PPI analysis. Clusters show significant overlap of PPI s of CA1 PNs DEGs, with few DEGs showing none or only 1 interacting partner. (B) Of the 1,566 DEGs in CA3 PNs, STRING found 1,482 proteins for PPI analysis. Clusters show significant overlap of PPI interactions of CA1 DEGs, with CA3 DEGs showing the lowest number of proteins with 1 or no interacting partners. (C) Of the 692 DGCs DEGs, 658 proteins were queried for PPI analysis. DGCs had the highest total number of proteins showing no or one interaction with the other proteins queried from the DEGs. [file Image_3.JPEG]

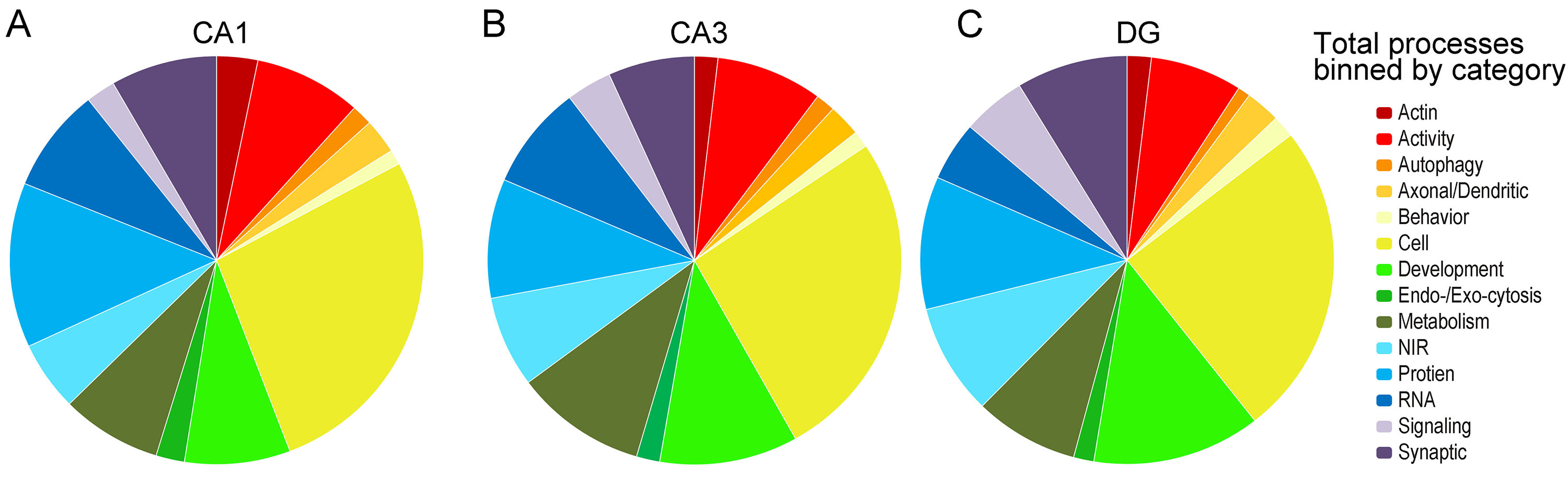

Supplement: SUPPLEMENTARY FIGURE S4 — GO analysis was employed to interrogate DEGs from the three hippocampal neuron populations. (A) Processes dysregulated in GO analysis in CA1 PNs were binned by category, with “protein” having relatively more dysregulation (percentage of total dysregulated pathways) compared to CA3 PNs or DGCs. (B) CA3 PNs showed similar dysregulation in terms of percentages of processes compared to CA1 PNs, with only slightly higher rates of metabolism and neurotransmitter, ions and receptor (NIR) processes dysregulated, although NIR had a higher percentage in DGCs compared to CA3 PNs. (C) DGCs showed relatively more dysregulated developmental processes, more than CA1 or CA3 and a moderately higher relative percentage of signaling processes were dysregulated. Signaling percentage was lowest in CA1 PNs, moderate in CA3 PNs, and highest in DGCs. [file Image_4.JPEG]
